# A Glutathione-Responsive Prodrug Nanosystem for Colorectal Cancer: Synergistic Co-Delivery of Paclitaxel and Curcumin to Combat Multidrug Resistance and Reinvent the Immunosuppressive Microenvironment

**DOI:** 10.34133/bmr.0399

**Published:** 2026-09-17

**Authors:** Yu Qiao, Shuai Yu, Chen Li, Dong-Dong Meng, Jia-Qi Niu, Dong-Run Yu, Xiao-Ming Wu, Hao-Xuan Wu, Wei-Tong Sun

**Affiliations:** ^1^ College of Pharmacy, Jiamusi University, Jiamusi City, Heilongjiang Province 154007, China.; ^2^ Heilongjiang Provincial Key Laboratory of New Drug Development and Pharmacotoxicological Evaluation, Jiamusi, Heilongjiang 154007, China.; ^3^ Xuzhou Xingchen Women and Children’s Hospital, Xuzhou Jiangsu Province, China.

## Abstract

The management of colorectal cancer is constrained by a self-perpetuating vicious cycle, driven initially by gut microbiota dysbiosis that triggers chronic inflammation, which in turn promotes the establishment of an immunosuppressive tumor microenvironment and multidrug resistance. We engineered a glutathione (GSH)-responsive prodrug micellar nanosystem (PSC/PTX) for the synergistic co-delivery of paclitaxel (PTX) and curcumin (CUR). In this nanoplatform, CUR was covalently conjugated to form a prodrug via disulfide linkages, while PTX was physically encapsulated within the micelles, ensuring the precise release of the 2 drugs in the tumor-specific microenvironment. The nanoplatform reverses P-glycoprotein-mediated drug efflux, prolongs the circulation time, improves the bioavailability, and enhances the accumulation at tumor sites of 2 drugs. This nanoplatform exerts multifaceted therapeutic efficacy. Initially, it reinstates the balance of intestinal microbiota by increasing the abundances of beneficial microorganisms (e.g., *Bacteroidetes*, *Eubacterium*, and *Bifidobacterium*) while decreasing those of harmful bacteria (e.g., *Bacteroides fragilis* and *Escherichia coli*). Additionally, it inhibits pro-tumor inflammatory factors by attenuating the levels of interleukin-6 (IL-6) and tumor necrosis factor-α (TNF-α), ultimately leading to a remodeling of the immunosuppressive tumor microenvironment (TME). This process involves boosting the infiltration of cytotoxic CD8^+^ T cells, decreasing the number of immunosuppressive regulatory T cells (Tregs), and repolarizing macrophages to the M1 phenotype (CD86^+^↑, CD206^+^↓). Collectively, this integrated and coordinated strategy breaks the self-sustaining vicious cycle of microbiota-driven, inflammation-fueled carcinogenesis and multidrug resistance via the synergistic modulation of chemotherapy sensitization, immunity, and the microbiota, thereby offering a novel and promising therapeutic modality for refractory colorectal cancer.

## Introduction

Colorectal cancer (CRC) is the third most common malignant tumor in the world, with the third highest incidence and mortality rate in the world, seriously threatening human health [1–3]. Currently, the primary clinical treatments for CRC include surgical resection, chemotherapy, gene therapy, and immunotherapy, with chemotherapy remaining the most widely used first-line treatment option [4,5]. However, most traditional chemotherapeutic drugs generally have problems such as poor targeting, rapid clearance in vivo, and the easy induction of multidrug resistance, which markedly compromise their therapeutic efficacy [6,7]. While the exact mechanisms underlying the pathogenesis of CRC remain incompletely understood, it has been established that dysbiosis of the gut microbiota plays a pivotal role in both the onset and advancement of CRC [8,9]. Additionally, research has shown that specific bacterial species, including *Fusobacterium nucleatum* and *Escherichia coli*, can intensify the inflammatory response by stimulating the Toll-like receptor 4 (TLR4)/nuclear factor κB (NF-κB) signaling pathway, compromise the integrity of the intestinal mucosal barrier, and form a vicious cycle of inflammation–carcinogenesis [10–12]. This vicious cycle substantially compromises the therapeutic efficacy of chemotherapy drugs and can even trigger the development of drug resistance [13,14]. Consequently, the exploration of safe and effective therapeutic agents or innovative treatments that can inhibit disease progression has become an urgent priority.

Paclitaxel (PTX) is a primary chemotherapeutic drug used in the clinical management of malignant tumors [15]. It operates by binding to β-microtubule proteins, thereby inhibiting cell proliferation by halting the cell cycle during the G_2_/M phase [16]. This interruption leads to mitotic dysfunction and ultimately triggers apoptosis in the affected cells [17]. However, CRC often exhibits insufficient intracellular PTX concentrations due to P-glycoprotein (P-gp) overexpression, preventing the drug from effectively stabilizing microtubule structures and arresting the cell cycle, thereby generating PTX resistance [18,19]. Additionally, single-agent treatment with PTX reduces the relative levels of probiotics, including *Bacteroidetes*, causing an imbalance in the gut microbiota and promoting the generation of inflammatory mediators [e.g., tumor necrosis factor-α (TNF-α) and interleukin (IL-6)], thereby indirectly diminishing its therapeutic effect on CRC [20]. Therefore, PTX is often administered in combination with other agents to reduce adverse effects and toxicity while avoiding the development of multidrug resistance and enhancing therapeutic efficacy. Curcumin (CUR) is a multidrug resistance inhibitor with a wide array of pharmacological effects [21]. CUR substantially suppresses P-gp expression and inhibits its substrate efflux activity; such intervention hinders the extracellular extrusion of PTX, efficiently reverses PTX-acquired chemoresistance in CRC cells, and recovers the chemosensitivity of neoplastic lesions to PTX-based regimens [22]. Moreover, by stimulating the canonical antioxidant Nrf2/ARE cascade and inhibiting overactivated NF-κB inflammatory signaling, CUR eliminates intracellular reactive oxygen species (ROS) and suppresses the release of core pro-inflammatory cytokines (e.g., TNF-α, IL-1β, and IL-6) [23]. Such dual modulation preserves intestinal epithelial barrier integrity, relieves PTX-evoked mucosal erosion and inflammatory infiltration, and thereby alleviates PTX-associated enteric damage and extraneous visceral toxicities in vivo [24]. In addition, CUR restores dysregulated gut microecology by elevating the relative abundance of beneficial commensals such as *Bifidobacterium*, whose reshaped intestinal communities trigger antitumor immune activation to synergistically potentiate the anti-colorectal carcinoma efficacy of PTX combination therapy [25]. However, both PTX and CUR suffer from poor water solubility and low stability. Therefore, a smart drug delivery system ought to be designed to enhance the solubility of PTX and CUR, diminish the toxicity of PTX, and enhance their antitumor efficacy.

In the past few years, advanced nanomedicine carriers—such as liposomes, nanoparticles, prodrug micelles, and dendritic macromolecules—have primarily focused on improving drug solubility, enhancing targeting, and increasing stability [26,27]. Polymeric prodrug micelles, prepared by the self-assembly of amphiphilic block copolymers, have attracted substantial interest due to their enhanced aqueous solubility, enhanced biocompatibility, mitigated adverse effects, enhanced permeability and retention (EPR) effect-facilitated passive tumor tissue retention, and extended in vivo circulation time [28]. Polymeric prodrug micelles achieve efficient co-loading and co-delivery of combination drugs by chemically bonding one drug to the carrier while physically encapsulating another, thereby forming prodrug micelles. These micelles enhance the loading capacity and in vivo stability of co-loaded drugs, prolong circulation time, improve targeting, and ultimately achieve highly efficient synergistic antitumor therapy [29,30]. Furthermore, polymeric prodrug micelles possess high design flexibility, with stimuli-responsive properties such as pH sensitivity [31], glutathione (GSH) sensitivity [32], and ROS responsiveness [33]. Among these, the GSH-response system utilizes the markedly higher GSH concentration in the tumor microenvironment (TME) compared to normal tissues (approximately 10 to 100 times that of normal cells) to trigger the specific disassembly of micelles at the tumor site and release the drug, thereby enabling targeted delivery and localized drug release through intelligent regulation. Currently, Poloxamer 188 (P188), also known as Pluronic F68, is extensively utilized in constructing drug delivery carriers owing to its low toxicity and excellent biocompatibility [34]. The outer layer of poly(ethylene oxide) (PEO) chains effectively evades recognition and clearance by the mononuclear phagocyte system (reticuloendothelial system) through steric hindrance, substantially extending the systemic circulation time of the drug and thereby enhancing drug accumulation at the tumor site [35]. These properties make P188 an ideal carrier material for constructing smart prodrug micelles.

**Fig. 1.**
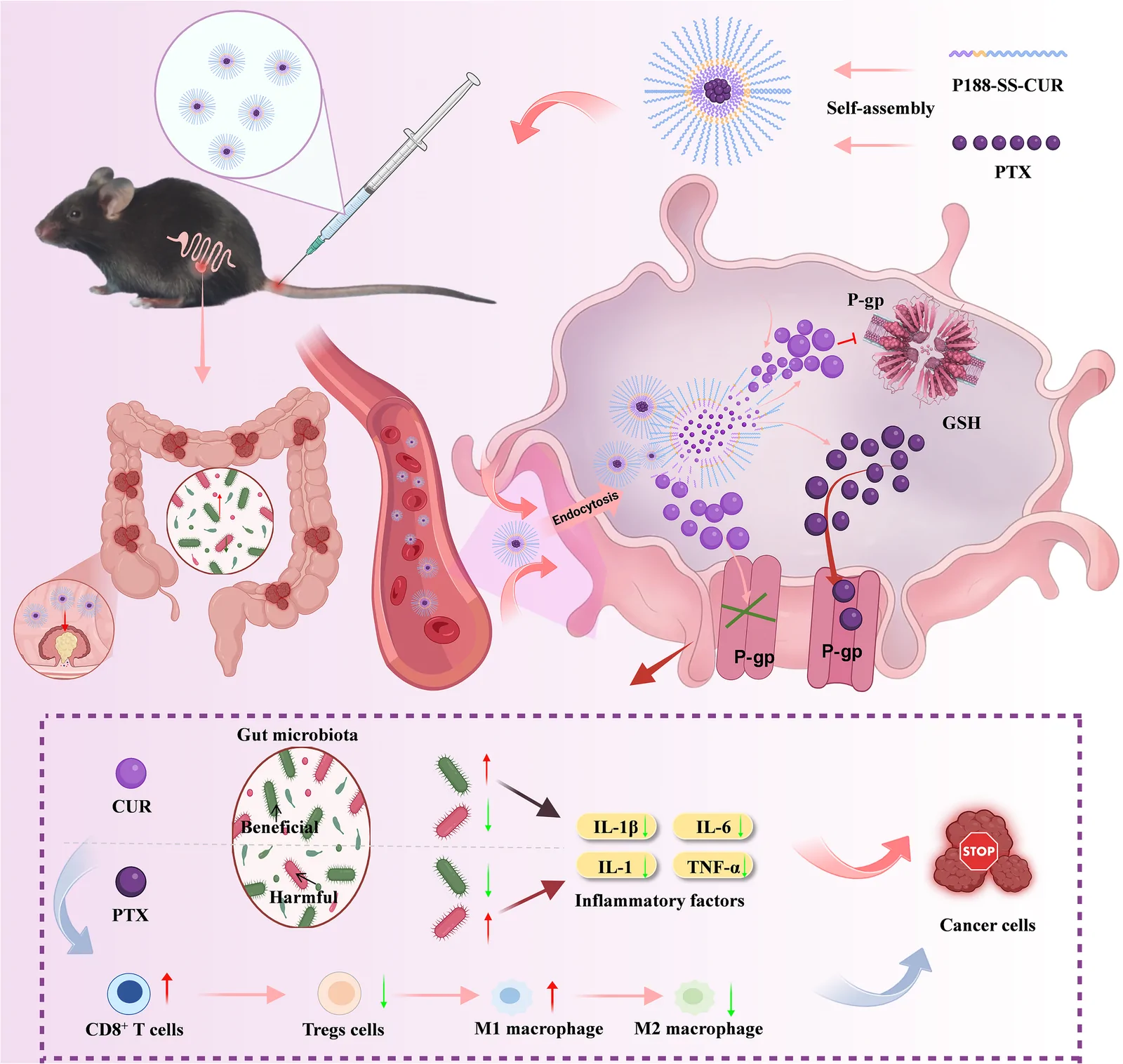
Fabrication protocol and self-assembly pathway of PSC/PTX.

In this study, the biocompatible P188 was selected as the hydrophilic segment, and CUR, which exhibits broad-spectrum pharmacological activity, was chosen as the hydrophobic segment. An amphiphilic block copolymer carrier, P188–SS–CUR, was constructed to respond to the TME through the introduction of reduction-sensitive disulfide bonds. On this basis, a PSC/PTX prodrug micelle system was constructed by encapsulating PTX to achieve dual-dimensional synergistic treatment. Specifically, the enhancement sensitivity mediated by disulfide bonds enhances targeted drug accumulation at tumor sites. Concurrently, CUR not only reverses PTX resistance by inhibiting P-gp expression but also blocks the vicious cycle of inflammation–carcinogenesis in CRC by reshaping the intestinal microbiota (e.g., augmenting the relative abundance of probiotic bacteria including *Lactobacillus*) and suppressing the level of inflammatory factor (e.g., IL-1β and IL-6). Furthermore, the combination of the 2 drugs synergistically promotes the skewing of macrophages toward the M1 subtype (CD86^+^↑, CD206^+^↓). This eventually establishes a multidimensional treatment mechanism of dual drug synergy–microbiota regulation–immune modulation. Meanwhile, we investigated the mechanisms underlying the synergistic effects of PTX and CUR in CRC treatment, providing feasible strategies and insights for targeted drug therapy for CRC. The fabrication protocol and self-assembly pathway of PSC/PTX are shown in Fig. 1.

## Materials and Methods

### Materials

Poloxamer 188, curcumin (98% purity), 3,3-dithiobispropionic acid, dimethyl sulfoxide (DMSO), paclitaxel (99% purity), sodium hydroxide, phosphate-buffered saline (PBS) buffer solution, reduced GSH, skimmed milk powder, ultra-sensitive enhanced chemiluminescence (ECL) kit, N,N,N′,N′-tetramethylethylenediamine (TEMED), ammonium persulfate, rabbit anti-human anti-P-gp antibody, rabbit anti-glyceraldehyde-3-phosphate dehydrogenase (GAPDH) polyclonal antibody, goat anti-rabbit horseradish peroxidase labeled secondary antibody, azoxymethane (AOM), and dextran sulfate sodium (DSS) were purchased from Merck Life Science Co. Ltd. 4-Dimethylaminopyridine (DMAP), *N*,*N*′-dicyclohexylcarbodiimide (DCC), pyrene, methanol (chromatographically pure), DMSO, 96-well cell culture plate, acetone, PBS buffer solution, glacial acetic acid, Tween 80, methanol (chromatographically pure), sodium dodecyl sulfate (SDS), potassium dihydrogen phosphate, and diethyl ether were bought from Shanghai Zhunteng Technology Co. Ltd. (Shanghai, China). Shanghai Aladdin Reagent Co. Ltd. (Shanghai, China) supplied acetone. Deuterated DMSO, methanol (chromatographic purity), acetonitrile (chromatographic purity), and dialysis bag [molecular weight cutoff (MWCO) 3,500 Da] glacial acetic acid were provided by Shanghai Junteng Technology Co. Ltd. (Shanghai, China). P188–SS–CUR (PSC), P188–CUR (PC), paclitaxel (PTX), and paclitaxel-resistant CRC cells SW480 were obtained from our laboratory. Fetal bovine serum (FBS), L15 medium, trypsin, penicillin–streptomycin solution, human CRC cells SW480, 4% paraformaldehyde, SDS-PAGE (polyacrylamide gel electrophoresis) buffer, 1.5 M tris-HCl (8.8), 1 M tris-HCl (6.8) trypsin digest, 4′,6-diamidino-2-phenylindole (DAPI) staining solution, l-glycine, and tris base were acquired from Shanghai Yuanpei Biotechnology Co. Ltd. (Shanghai, China). Cell Counting Kit-8 (CCK-8) Cell Proliferation Assay Kit was provided by Shanghai Yuanxin Biotechnology Co. Ltd. (Shanghai, China). Dialysis bag (MW = 3,500 Da), sterile pH 7.4 PBS buffer solution, methanol, cell lysate (radioimmunoprecipitation assay), SDS, protease inhibitor phenylmethylsulfonyl fluoride (PMSF), DMSO, Nile Red, bicinchoninic acid (BCA) protein quantification kit, pre-stained protein marker, and Tween 20 were bought from Thermo Fisher Scientific. Corning (China) supplied the 6-well cell culture plate. Paraformaldehyde (4%) was obtained from Biosharp Technologies Co. Ltd. (Anhui, China). Hematoxylin staining solution and eosin staining solution were acquired from Biyuntian Biotechnology Co. Ltd. IL-6 kit, TNF-α kit, and IL-1β kit were purchased from Nanjing Jiancheng Technology Co. Ltd.

### Cells and animals

The human CRC cell lines SW480 and NCM460 (Shanghai Yuanpei Biological Technology, China) were cultured in RPMI 1640 medium (Gibco, USA) supplemented with 10% FBS and 1% penicillin–streptomycin (Biyuntian Biotechnology, China) at 37 °C in a 5% CO₂ humidified incubator.

Male C57BL/6 mice (4 to 6 weeks old, 20 ± 2 g) and Sprague–Dawley rats (males, body weight: 200 to 220 g) were obtained from Liaoning Changsheng Biotechnology Co. Ltd. (China). All animal experiments were conducted in accordance with protocols approved by the Jiamusi University Laboratory Animal Management Committee (JMSU-2023110804 and JDYXY-2026007).

### Preparation and characterization of PSC/PTX

3,3′-Dithiodipropionic anhydride (DTDPA) was prepared by refluxing 3,3′-dithiodipropionic acid (DTPA) (2.0 g, 9.5 mmol) in acetyl chloride (6 ml) at 65 °C for 2 h, followed by rotary evaporation and vacuum filtration with cold diethyl ether. For P188-SS-COOH, P188 (4.0 g, 0.476 mmol) and DTDPA (0.10 g, 0.52 mmol, molar ratio 1:1.1) were dissolved in DMSO (4 ml), followed by addition of DMAP (0.80 g) and DCC (0.80 g) in DMSO (3 ml). The mixture was stirred at room temperature for 48 h and then purified by dialysis (MWCO 3,500 Da) against ultrapure water for 48 h and lyophilized. PSC was obtained by activating P188-SS-COOH (4.0 g, 0.476 mmol) with DMAP/DCC (0.90 g each) in DMSO (12 ml) for 2 h, dissolving CUR (0.70 g, 1.9 mmol, molar ratio 1:4) in DMSO (6 ml) with stirring in the dark for 2 h, then combining and stirring in the dark at room temperature for 48 h, followed by dialysis and lyophilization using the same procedure as for the previous compound. P188-COOH was afforded by reacting P188 (5.0 g, 0.595 mmol) with succinic anhydride (0.27 g, 2.70 mmol, molar ratio 1:4.5) in 1,4-dioxane (20 ml) at room temperature for 24 h using DMAP (0.14 g) and triethylamine (TEA) (0.25 ml), followed by dialysis and lyophilization using the same procedure as described above. PC was prepared following the same procedure as PSC, with P188-COOH (4.0 g, 0.476 mmol), DMAP/DCC (0.10 g each), and CUR (0.70 g, 1.9 mmol, molar ratio 1:4) in DMSO (12 ml for activation, 6 ml for CUR), with identical reaction and purification conditions.

PSC/PTX prodrug micelles were prepared by co-dissolving PTX (1.9 mg) and PSC (20 mg) in methanol (10 ml) under sonication. After rotary evaporation at 40 °C to yield a film, the product was hydrated with purified water (10 ml) at 39 °C for 1 h with stirring. The resulting solution was then filtered via a 0.22-μm membrane to complete the synthesis.

The PSC/PTX prodrug micelles were characterized for their particle size, zeta potential, and polydispersity index (PDI) by dynamic light scattering (DLS), and for their morphology by transmission electron microscopy (TEM; HT7700, Hitachi). Thermal properties were analyzed using differential scanning calorimetry (DSC; STA409PC, Hengjiu). Encapsulation efficiency (EE%) and drug loading (DL%) were determined via ultrafiltration centrifugation and calculated according to the following formula:

$$\mathrm{EE}\%=\left(1-C_{\mathrm{PTX}}/C_{\text{total}}\right)\times 100\%$$ (1)

$$\mathrm{DL}\%=\left(W_{\mathrm{PTX}}/W_{\text{total}}\right)\times 100\%$$ (2)

### Stability study

DLS was employed to evaluate the plasma stability and storage stability of PSC/PTX prodrug micelles. For storage stability, the prepared PSC/PTX prodrug micelles were stored at 4 and 25 °C for 30 d, and these samples were measured and recorded at 1, 3, 7, 15, and 30 d. For plasma stability, PSC/PTX prodrug micelles were placed into the PBS solution containing 10% serum. Following incubation with shaking at 37 °C for 1 d, the particle size was assessed at 0, 4, 8, 12, and 24 h, respectively.

### The hemolysis assay

The mouse blood was collected into heparinized centrifuge tubes, mixed with physiological saline, centrifuged for 10 min, and allowed to stand before removal of the supernatant. Next, the red blood cells were redispersed by rubber-tipped dropper after the precipitate was removed using 0.9% sodium chloride. This process was repeated until the upper-layer solution turned colorless. Finally, the red blood cells that were compacted in the lower layer were introduced to 10 ml of normal saline to produce a 2% red blood cell suspension. After transferring the suspensions to centrifuge tubes, they were mixed with 3 ml of PSC/PTX prodrug micelle solutions (1, 2, 4, 8, and 16 mg/kg). Positive (3 ml of saline) and negative (3 ml of distilled water) controls were run in parallel. All tubes were incubated at 37 °C for 4 h and centrifuged at 3,000 rpm for 10 min at 4 °C, and the hemolysis rate was determined as follows:

$$\begin{array}{cc} \text{Hemolysis rate} & =\left(\text{Asample}-\text{Anegative}\right)\\ & -\left(\text{Apositive}-\text{Anegative}\right)\times 100\% \end{array}$$ (3)

### In vitro release of PSC/PTX prodrug micelles and PC/PTX prodrug micelles

For the elucidation of the reduction-sensitizing effect of drug-loaded micelles, PBS (pH 7.4, 0.01 M ionic strength) solutions containing GSH at 0 and 10 mM were added to drug-loaded micelles and stirred at 50 rpm under constant temperature conditions (37 ± 0.5 °C) for 24 h, and mean particle diameter was analyzed by DLS.

The dynamic dialysis method was utilized to evaluate the in vitro release of micelles composed of PC/PTX prodrug, PSC/PTX prodrug, and free drugs. Each type of solution, including PTX, CUR, PC/PTX, and PSC/PTX, was placed into dialysis bags (MW = 3,500 Da), respectively. Subsequently, free drug (PTX and CUR) solutions and drug-loaded micelles (PC/PTX and PSC/PTX) were subjected to dialysis under distinct conditions. The former used pH 7.4 PBS with 0.5% Tween 80. The latter used the same buffer supplemented with 10 mM GSH. All dialyses were performed at 37 °C with 100 rpm agitation. Samples (5 ml) were taken at scheduled times, and the cumulative release of PTX and CUR was calculated to generate the release curves.

### Cell culture and determination of resistance

The PTX-resistant cell lines SW480/PTX were established by the method of induction with increasing drug concentrations. Firstly, 5 ml of SW480 cells was seeded into 25-cm^2^ culture flasks at a density of 1 × 10^4^ cells/ml. Following the stable growth of cells, they were incubated with 0.1 μg/ml of PTX. The culturing procedure was maintained until the cells were capable of growing normally at this concentration. After undergoing 2 to 3 passages, the concentration of paclitaxel was gradually increased. CRC cells that were stably growing in a medium containing 25.0 μg/ml PTX were regarded as PTX-resistant cells. The determination of the half-maximal inhibitory concentration (IC_50_) for the cells was accomplished using the CCK-8 assay and GraphPad Prism 10 software. Drug resistance index (RI) of SW480/PTX = IC_50_ (SW480/PTX)/IC_50_ (SW480). Once RI was greater than 5, the cultivation of drug-resistant cells was finalized.

### Cellular uptake

The SW480/PTX cells were cultured in 6-well plates until they reached a stable growth phase, at which point the old medium was exchanged for a medium containing the drug. After culturing for 90 min, the cells were rinsed 3 times with PBS and fixed with 4% paraformaldehyde. Then, the cells were treated using a staining medium and the uptake behavior of the cells was observed using an inverted fluorescence microscope (IFM) under dark conditions. DAPI: nucleus; green: CUR can emit green light; red: Nile Red (Nile Red is a replacement for PTX).

### Cell cytotoxicity

#### Cytotoxicity assays of blank vector

The impact of PC and PSC empty vectors on the survival rates of NCM460, SW480, and SW480/PTX cells was examined using the CCK-8 assay. After adding the medium containing the blank vector to the 6-well plate, the incubation was set for 48 h with 6 concentration gradients (0, 1, 10, 35, 70, and 100 μg/ml). Then, the CCK-8 solution (10 μl) was added and incubated for 2 h. The calculation of cell viability is carried out using the following formula:

$$\text{Cell viability rate}\;\left(\%\right)=\left[\left(\mathrm{As}-\mathrm{Ab}\right)/\left(\mathrm{Ac}-\mathrm{Ab}\right)\right]\times 100\%$$ (4)

#### Cytotoxicity assays of drug-loaded micelle PTX/PC and PTX/PSC

The determination method of drug-loaded micelles was consistent with that of blank carriers. The following groups were established: free paclitaxel (PTX) group, PTX/CUR (1.3:1) group, PC/PTX group, and PSC/PTX group. For each of these groups, the mass concentrations of PTX were established at 0, 1, 2, 4, 12, 24, and 40 μg/ml, respectively. Additionally, control group and blank group were also set up. The viability of the cells was assessed via the formula presented below:

$$\text{Cell inhibition rate}\;\left(\%\right)=1-\text{cell viability rate}\;\left(\%\right)$$ (5)

### Wound healing assay

The inhibitory effects of different drug groups (CUR, PTX, CUR/PTX, PC, PSC, PC/PTX, and PSC/PTX) on drug-resistant cell migration were examined by wound healing experiments. Drug-resistant cells were added to 6-well plates for incubation. The sterile pipette tips were utilized to create scratches within the cellular layer, which were rinsed with PBS to eliminate any dislodged cells. Thereafter, the culture medium containing the drugs was added. The fluorescent inverted microscope was applied to capture images of the different groups.

### Western blot

The detection was performed using the BCA protein detection kit (Solarbio, Beijing, China) method (Solarbio, Beijing, China). The total protein of SW480/PTX cells was extracted for SDS-PAGE separation, transferred to polyvinylidene difluoride (PVDF), and incubated with primary antibody (overnight at 4 °C) and secondary antibody (room temperature for 1 h) after 5% skimmed milk powder, and finally developed color.

### Pharmacokinetic studies

Sprague–Dawley rats (males, body weight: 200 to 220 g) were randomly assigned to 3 groups. Each group was given PSC/PTX prodrug micelles (10 mg/kg CUR, 10 mg/kg PTX) through the tail vein, along with preparations containing free CUR and PTX. Blood samples (500 μl) were harvested from the posterior orbital venous plexus of rats at various time points (0.083, 0.25, 0.5, 1, 2, 4, 8, 12, 24, and 48 h). Heparinized tubes were used to place all samples, which were then centrifuged at a speed of 3,000 rpm for 10 min to collect the supernatant. At 4 °C, the collected plasma samples underwent rapid centrifugation at 12,000*g* for 10 min, and the resulting supernatant (plasma) was kept at −80 °C until further analysis. At the experimental endpoint, rats were deeply anesthetized with inhaled isoflurane (3% to 5% for induction and 1.5% to 2.5% for maintenance) and euthanized by cervical dislocation. The heart, lungs, liver, kidneys, brain, and spleen were immediately collected and homogenized in ice-cold physiological saline. Finally, quantitative analysis was conducted utilizing high-performance liquid chromatography (HPLC). The HPLC system utilized in this study was the Agilent 1260 Infinity II (Agilent Technologies, Santa Clara, CA, USA), featuring a reversed-phase C18 column (Agilent ZORBAX SB-C18, dimensions of 4.6 × 250 mm, and a particle size of 5 μm) alongside a ultraviolet–visible detector. The mobile phase consisted of ultrapure water and acetonitrile in a ratio of 65:35 (v/v), flowing at a rate of 1.0 ml/min, while the temperature of the column was kept at 30 °C. Detection wavelengths for PTX and CUR were established at 227 and 425 nm, respectively, with an injection volume of 20 μl. Furthermore, at the study endpoint, major organs (heart, liver, spleen, lung, and kidney) were harvested for hematoxylin and eosin (H&E) staining, and serum levels of alanine aminotransferase (ALT), aspartate aminotransferase (AST), blood urea nitrogen (BUN), and creatinine (CREA) were measured.

### In vivo therapeutic efficacy

C57BL/6 mice were administered to the AOM-DSS chemically induced CRC model over 63 d, followed by a 30-d drug administration period. Briefly, mice were allocated into 7 groups (10 mice in each group) via ear tagging: blank control, model, PTX, CUR, PTX/CUR, PC/PTX, and PSC/PTX groups. Except for the control group, each group was intraperitoneally injected with AOM (10 mg/kg). After 1 week, they were provided with a 2% DSS solution to drink for 7 d, after which they were given drinking water for 2 weeks (a 21-d cycle). This cycle was reiterated 3 times to complete model induction. During the treatment phase, mice in treatment groups received intravenous injections of respective drugs (10 mg/kg) every 2 d. Throughout the experiment, general conditions (coat color, activity, mental state, fecal consistency, and anal status) were monitored daily, and body weight was recorded weekly. Following completion of the in vivo efficacy assessment, tumors were surgically excised, weighed, and subjected to H&E staining to evaluate histopathological characteristics. Detailed descriptions of the sample size allocation across different experimental modules are as follows:

For the in vivo antitumor and anti-inflammatory efficacy study, 10 mice per group were initially assigned for the efficacy evaluation. These animals were used throughout the entire experiment for dynamic body weight monitoring, colon length measurement at the experimental endpoint, tumor burden assessment, and histopathological analysis of colon tissues. For animals that either died spontaneously during the prolonged AOM/DSS modeling period or were humanely euthanized when they became moribund from severe colitis, all data were processed strictly following the intention-to-treat (ITT). Specifically, for body weight measurements, all historical body weight data from surviving time points prior to death were fully retained and included in the longitudinal repeated-measures statistical analysis, with no new data recorded after death. Endpoint anatomical parameters: For moribund euthanized or spontaneously deceased mice, immediate dissection and tissue collection were performed on the day of death, with prompt measurement of colon length, quantification of tumor number and burden, and preparation of pathological sections, all of which served as the endpoint data for each individual animal. In contrast, mice that survived to the predefined experimental endpoint were dissected and examined at a unified termination time point using the same standardized procedures. Thus, every one of the 10 initially enrolled mice per group contributed a complete set of observational data with no missing values, and the final statistical sample size for all 4 endpoint indicators remained at *n* = 10 per group, ensuring robust statistical validity.

The pharmacokinetic analysis was performed using an independent, parallel cohort of rats that was completely separate from the 10 animals used for efficacy evaluation. This design was necessary because pharmacokinetic (PK) assessment requires serial blood sampling from the retro-orbital plexus at multiple time points (0.083, 0.25, 0.5, 1, 2, 4, 8, 12, 24, and 48 h post-administration). Frequent blood collection would compromise the circulatory system, disrupt intestinal inflammatory homeostasis, and interfere with AOM/DSS-induced colitis and tumorigenesis, making it incompatible with the efficacy study. Therefore, we pre-established a separate cohort of 6 rats per group exclusively for pharmacokinetic profiling. This sample size was determined based on our preliminary PK experiments and represents a reasonable number of biological replicates. Consequently, the PK data (*n* = 6 per group) do not involve sample reuse or conflict with the main efficacy study.

The enzyme-linked immunosorbent assay (ELISA) analysis required fresh, nonhemolyzed serum samples collected at the experimental endpoint. During the 3 cycles of AOM/DSS treatment, some mice died naturally due to severe rectal bleeding, sustained and pronounced body weight loss, or excessive tumor burden. Moribund individuals were humanely euthanized in strict accordance with our institutional animal ethics guidelines. For these deceased animals, matched serum samples at the unified experimental endpoint could not be collected, as they did not survive to the scheduled termination time point. Among the remaining surviving mice, cases of hemolysis during blood collection or tissue degradation during processing similarly precluded their use for ELISA quantification. Ultimately, only 5 mice per group remained viable with successfully collected and adequately preserved serum samples meeting all quality control requirements. Accordingly, the cytokine ELISA assay was conducted with *n* = 5 biological replicates per group.

Considering the relatively high cost of 16*S* ribosomal RNA (rRNA) high-throughput sequencing, a sampling scheme was predefined prior to any data analysis, with the aim of maintaining statistical representativeness while reasonably containing experimental expenditure. Specifically, we applied simple random sampling to select 3 of the 5 qualified mice per group for microbiota profiling. Fecal samples (colon contents) from these randomly selected individuals were subjected to sequencing analysis. Importantly, the selection process was entirely randomized and involved no manual bias or preference for favorable experimental outcomes.

### Enzyme-linked immunosorbent assay

First, the sample to be tested and the test kit were removed and left at room temperature for a period of time. Fifty microliters of different concentrations of standards was added to each standard well. Subsequently, the sample (10 μl) to be tested and sample dilution (40 μl) were pipetted to the wells. One hundred microliters of the detection antibody [labeled with horseradish peroxidase (HRP)] was pipetted into the wells of standard and the wells of the sample to be tested. Following incubation at 37 °C for 1 h, the liquid was discarded and the washing solution was added for plate washing. Subsequently, an appropriate number of substrates was introduced into every well and treated for 15 min. The absorbance values were determined after addition of stop solution (50 μl).

### Microbiological analysis of intestinal flora

The intestinal tracts of mice were dissected to extract fecal matter, which was then placed into sterile freezing vials and frozen at −80 °C for subsequent analysis and dispatch for examination. Subsequently, the changes in the gut microbiota across different sample groups were detected and analyzed using a high-throughput sequencing platform.

### Immunohistochemical analysis

To analyzed the polarization phenotypes of CD8^+^ T cells (anti-CD8α), regulatory T cells (Tregs) (anti-Foxp3 antibody), and macrophages (pan-macrophage marker CD68 and M1/M2 markers CD86 and CD206, respectively), we analyzed colorectal tumor tissues via immunohistochemistry (IHC). After antigen repair, the sections were stained with conventional IHC (primary antibody 4 °C overnight, HRP secondary antibody, 3,3′-diaminobenzidine (DAB) chromogenic, hematoxylin counterstaining). Macrophage phenotyping was achieved by multiplex immunofluorescence, using anti-CD68 (AF594), anti-CD86 (AF488), and anti-CD206 (AF647) antibodies to label pan-macrophages and M1 and M2 types, respectively.

### Statistical analyses

The experimental data were analyzed with GraphPad Prism 9.0 software. The mean ± SD was employed to express the results, and one-way analysis of variance (ANOVA) was used to evaluate the variances among the groups. When the *P* value was less than 0.05 (*P* < 0.05), it was considered statistically significant.

## Results and Discussion

### Synthesis and characterization of PSC and PC

**Fig. 2.**
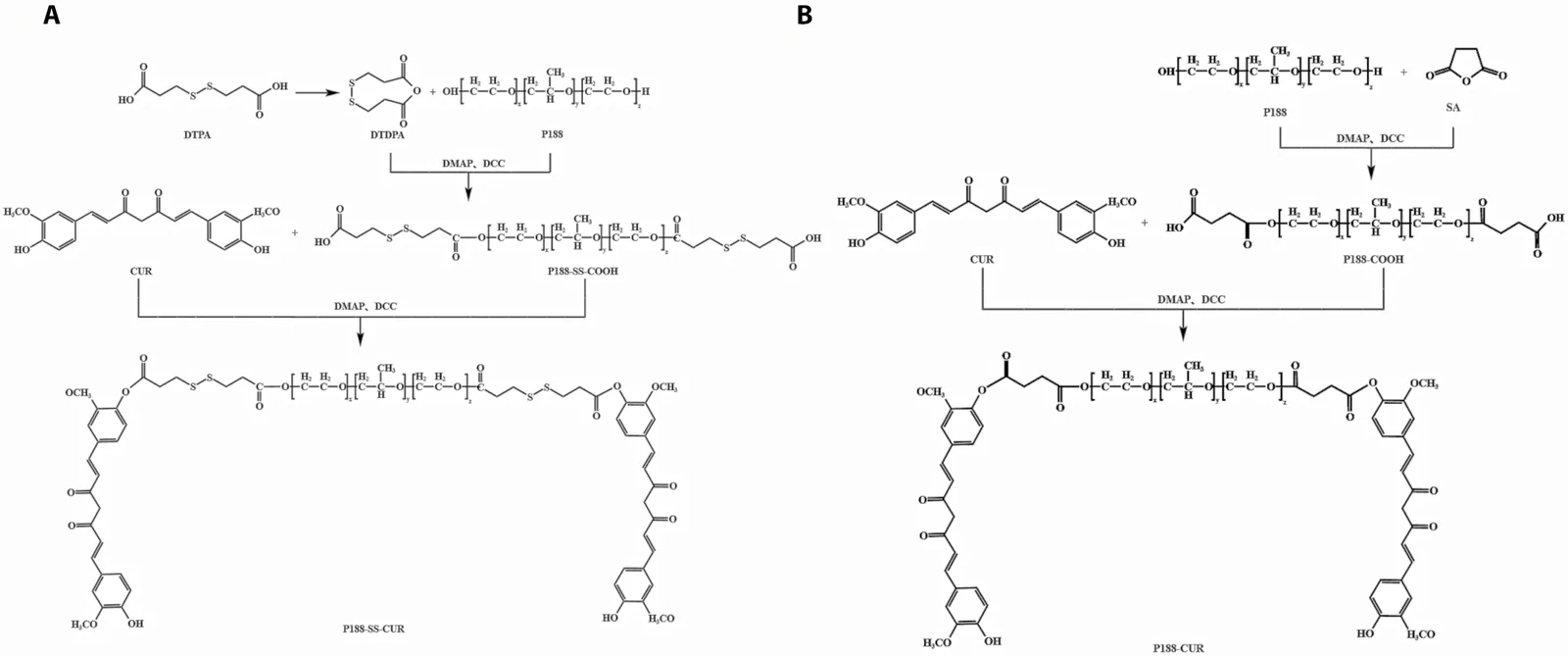
Synthesis route for (A) PSC and (B) PC.

**Fig. 3.**
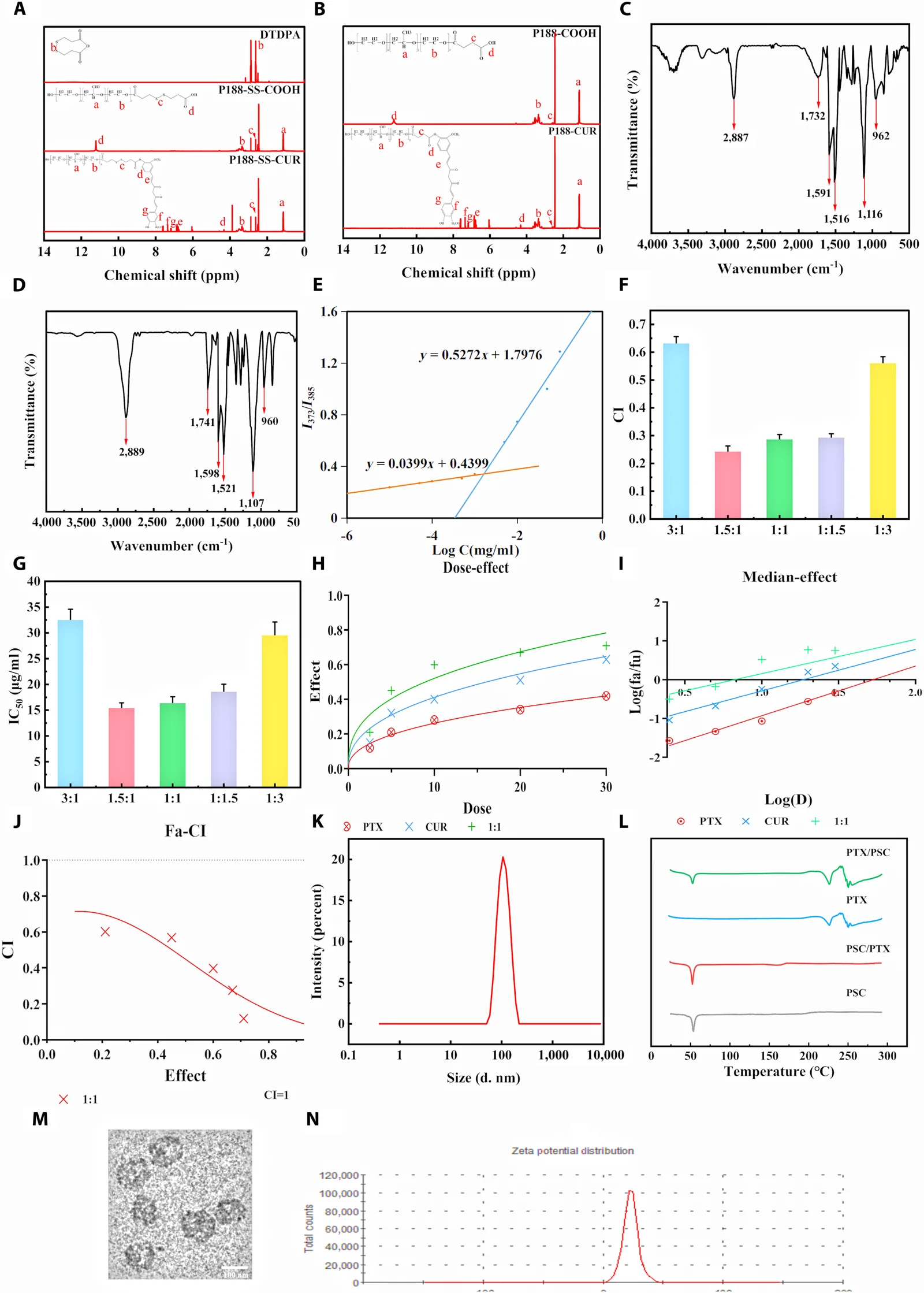
Detection of carrier synthesis and correlation characterization of nanoparticles. ^1^H NMR spectra of DTDPA, P188-SS-COOH, (A) PSC and P188-COOH and (B) PC were added in DMSO-d6 and measured by 400-MHz ^1^H NMR spectrometry. FTIR spectra for (C) PSC and (D) PC were obtained (Spectrum100 spectrometer, KBr pellet method) over 4,000 to 500 cm^−1^ at a resolution of 0.5 cm^−1^. (E) Critical aggregation concentrations of PSC prodrug micelles (excitation and emission wavelength slit width: 5 nm, sensitivity: 3, excitation wavelength: 334 nm, scanning wavelength: 200 to 600 nm). (F) CI values and (G) IC_50_ values for different proportions of PTX and CUR in SW480/PTX cells; (H) volume–effect relationship curves, (I) median–effect curves, and (J) Fa–CI curves of CUR and PTX in SW480/PTX cells. All experiments were performed with a cell density of 1 × 10^4^ cells/well and held for 48 h. (K) Particle size diagram of PSC/PTX prodrug micelles (measured by Zetasizer Nano ZSE at 25 °C). (L) DSC profiles of PSC/PTX micelles (heating rate: 10 °C/min, scanning range: 25 to 300 °C). (M) Transmission electron micrograph of PSC/PTX micelles (scale bar, 100 nm). (N) Zeta potential distribution of PSC/PTX prodrug micelles (used by Zetasizer Nano ZSE at 25 °C).

The synthesis routes of PSC and PC were shown in Fig. 2A and B. The chemical structures of PSC and PC were validated by ^1^H nuclear magnetic resonance (NMR) analysis. Figure 3A displays the ^1^H NMR spectrum of the PSC polymer. The characteristic peak associated with the carboxyl group (-COOH) was absent in the DTDPA spectrum, indicating that DTDPA was successfully synthesized. In the spectrum of P188-SS-COOH, the peaks at 1.13 and 3.35 ppm corresponded to the characteristic (-CH_2_) peaks between -CH_3_ and PEO in the poly(propylene oxide) (PPO) segment of P188. Additionally, the characteristic peaks at 2.62 and 11.2 ppm were attributed to (-CH_2_-CH_2_-S-S-CH_2_) and (-COOH) of dithiodipropionic acid, respectively. These results confirmed the successful synthesis of P188-SS-COOH. As an overview of the PSC spectral information, both 1.16 and 3.34 ppm corresponded to the characteristic peaks of P188. The characteristic peak of 2,2'-dithiodipropionic acid was observed at 2.62 ppm, while the peak at 4.3 ppm belonged to the methylene group (-CH_2_) linked to the ester group. The signals at 6.74, 7.35, and 7.19 ppm were signaling peaks of CUR. The above results indicated that PSC was successfully synthesized. The ^1^H NMR spectrum of the PC polymer was confirmed in Fig. 3B. A new signal appeared at 11.2 ppm, indicating that succinic anhydride was successfully conjugated to the hydroxyl groups of P188, thereby confirming the synthesis of P188-COOH. The signal peaks at 2.53 and 4.35 ppm correspond to succinic acid and methylene (-CH_2_) groups linked to the ester moiety, respectively. The peaks at 6.76, 7.41, and 7.17 ppm were characteristic peaks of CUR benzene ring within the 6- to 8-ppm range. These results demonstrated that PC was successfully synthesized.

The Fourier transform infrared (FTIR) spectrum of PSC indicated the formation of the compound (Fig. 3C). The absorption peaks at 2,887 and 480 cm^−1^ were related to -C–H and -C–O in P188, respectively. The signal peaks at 1,591 and 1,516 cm^−1^ were the stretching vibration peaks of C=C and C–C–C in the benzene ring of CUR, respectively. The absorption at 962 cm^−1^ resulted from the out-of-plane C–H vibration of the aromatic ring in CUR. The peak at 1,732 cm^−1^ corresponded to the stretching vibration of the ester bond (C=O). The above results demonstrated that PSC was successfully synthesized. The structure of PC was demonstrated by FTIR (Fig. 3D). The peaks at 2,889 and 1,107 cm^−1^ were the characteristic peaks of -C–H and -C–O in P188, respectively. Notably, the characteristic peaks at 1,598, 1,521, and 960 cm^−1^ were the stretching vibration of C=C, the in-plane vibration of C–C–C, and the out-of-plane bending vibration peak of aromatic hydrocarbon bond (C–H) in CUR, respectively. Additionally, the absorption peak at 1,741 cm^−1^ corresponds to C=O in the ester bond. The above results confirmed that PC was successfully synthesized.

### Determination of the critical aggregation concentration

The critical aggregation concentration (CAC) of the polymer (PSC) was measured by pyrene fluorescent probe technology. The ratio of *I*_373_/*I*_384_ varied with the increase in polymer concentration (Fig. 3E). The CAC value for PSC was calculated to be 1.6 × 10^−3^ mg/ml based on the intersection of 2 linear segments. The result indicated that PSC polymers can spontaneously self-assemble into stable micelles at very low concentrations, providing a robust foundation for subsequent drug encapsulation.

### Synergistic effect

To identify the most effective synergistic inhibition ratio of PTX and CUR on SW480/PTX cells, we integrated the CCK-8 assay with the Chou–Talalay approach for analyzing the data. As shown in Fig. 3F to J, the IC_50_ values, combination index (CI) values, median effect curves, dose–effect curves, and Fa–CI curves were calculated using Calcusyn software. Notably, the IC_50_ value was minimized at a PTX:CUR ratio of 1.5:1, indicating the strongest synergistic interaction. Furthermore, the Fa–CI curve for SW480/PTX cells demonstrated CI < 1 across all tested ratios, confirming consistent synergy between PTX and CUR. Within the PTX:CUR ratio range of 1.5:1 to 1:1.5, the CI values remained below 0.3, indicating an exceptionally strong synergistic effect. Based on these findings, the optimal synergistic ratio range for PTX and CUR was identified as 1.5:1 to 1:1.5.

### Preparation and characterization of PSC/PTX prodrug micelles

DLS was employed to evaluate the particle size, zeta potential, and PDI of the PSC/PTX prodrug micelles. The EE% and DL% of the PSC/PTX prodrug micelles were measured as 87.16 ± 0.65% and 7.13 ± 0.07%, respectively. The mean particle size, PDI, and zeta potential of the PSC/PTX prodrug micelles were 105.7 ± 1.84 nm, 0.117 ± 0.12, and −29.13 ± 0.07 mV, respectively (Fig. 3K and N). Additionally, the morphology of PSC/PTX prodrug micelles was characterized by TEM (Fig. 3M), revealing a quasi-spherical shape with a smooth surface, relatively uniform particle size, and favorable dispersity.

DSC was performed on samples of PSC/PTX, PTX, the physical mixture (PTX/PSC), and the PSC polymer (Fig. 3L). The characteristic peak of PTX appeared at 225 °C, while the PSC polymer exhibited a distinct endothermic peak at 53 °C, with no overlap between the 2. Importantly, in the DSC curve of PSC/PTX, the characteristic PTX peak was absent, and only the peaks corresponding to the PSC polymer were detected. These results indicated that PTX was successfully incorporated into the micelles, further confirming the successful preparation of PSC/PTX prodrug micelles.

### Stability studies

**Fig. 4.**
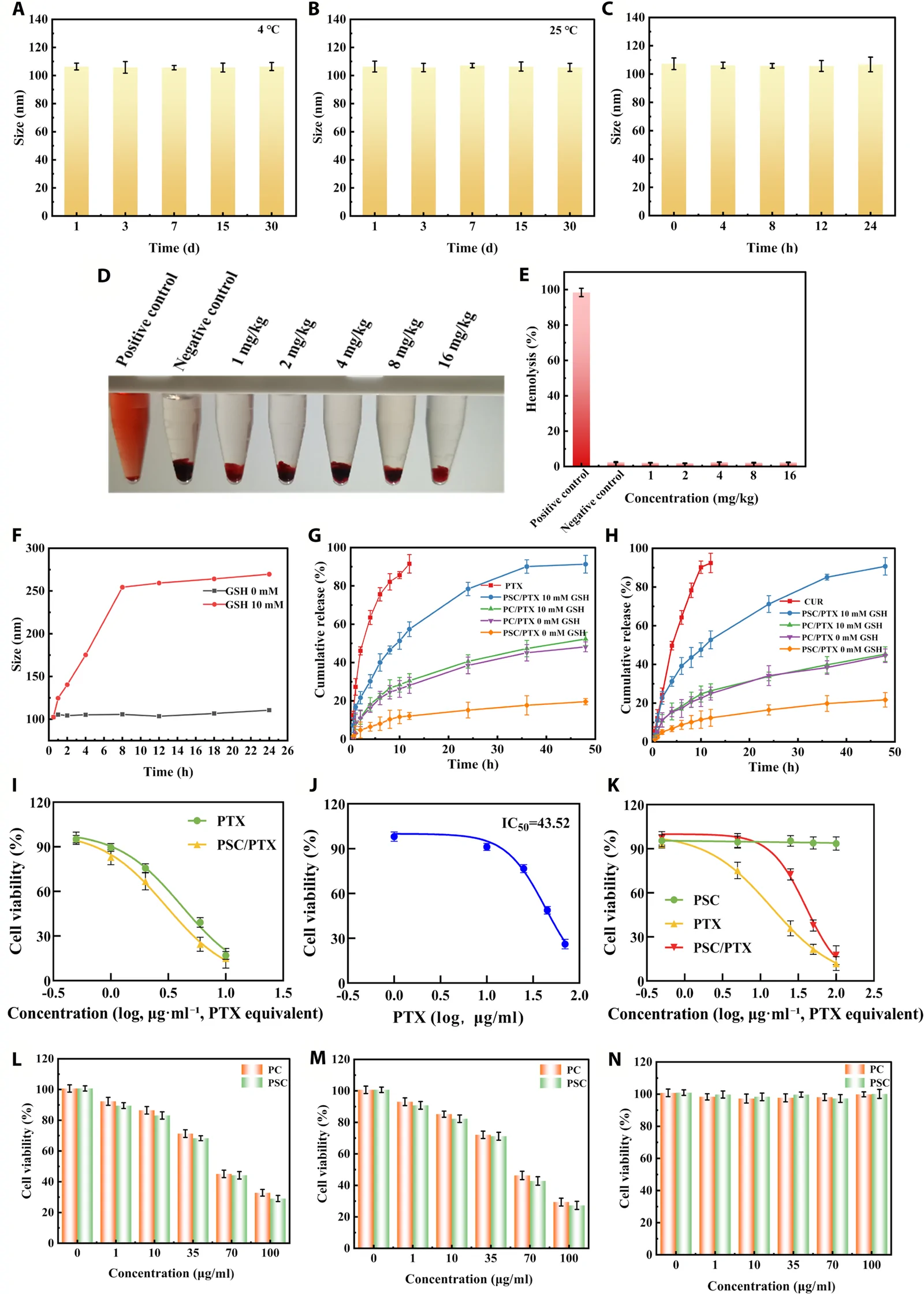
Study on safety, stability, and drug release of PSC/PTX prodrug micelles. PSC/PTX particle size at (A) 4 °C for 30 d and (B) (25 ± 0.5) °C for 30 d. (C) Plasma stability of the PSC/PTX prodrug micelles at 37 °C after holding for 24 h. (D) Hemolysis phenomenon and (E) hemolysis rate of PSC/PTX prodrug micelles at different concentrations (held at 37 °C for 4 h, centrifugation: 4 °C, 3,000 rpm, 10 min; positive control group: pure water, negative control: saline). (F) PSC/PTX prodrug micelle particle size changes at 0 and 10 mM GSH. In vitro release behavior of (G) PTX and (H) CUR from PSC/PTX and PC/PTX prodrug micelles at various time points in 0 mM GSH and 10 mM GSH administration (pH 7.4 PBS solution + 0.5% Tween 80 + 10 mM GSH and pH 7.4 PBS solution + 0.5% Tween 80). IC_50_ values of PTX and PSC/PTX on SW480 (I) and IC_50_ values of PTX on SW480/PTX cells (J) (cell density: 1 × 10^4^, PTX concentration: 0.2 to 5.0 μg/ml, PSC/PTX concentration: 1 to 100 μg/ml). (K) IC_50_ values of PSC, PTX, and PSC/PTX on NCM460 (cell density: 1 × 10^4^, PTX concentration: 0.2 to 5.0 μg/ml, PSC concentration: 1 to 100 μg/ml, PSC/PTX concentration: 1 to 100 μg/ml). Effects of PC and PSC on cell survival rates of (L) SW480, (M) SW480/PTX, and (N) NCM460 cells at different concentrations after holding for 48 h at 37 °C (PC and PSC concentration: 1 to 100 μg/ml) (*n* = 3, compared with PTX, *P < 0.05, **P < 0.01).

The storage stability and plasma stability of PSC/PTX prodrug micelles were assessed by detecting changes in their particle size (Fig. 4A to C). The particle size of PSC/PTX prodrug micelles showed no marked change at 4 and 25 °C, indicating superior storage stability. Furthermore, the particle size remained relatively stable after 24 h of incubation in a simulated blood environment, demonstrating excellent plasma stability. These stability profiles ensure that the micelles can effectively preserve their drug-loading capacity and targeting ability, laying a reliable foundation for subsequent in vivo antitumor applications.

### Hemolysis assay

The hemolysis rate of PSC/PTX prodrug micelles was determined by hemolysis assay (Fig. 4D and E). The hemolysis rates of PSC/PTX prodrug micelles at different concentrations consistently remained below 3%, indicating that PSC/PTX prodrug micelles solutions exhibit excellent blood compatibility, similar to the negative control (saline). By contrast, the positive control (pure water) showed hemolysis due to the rupture of erythrocytes caused by marked water absorption. Collectively, these results indicated that PSC/PTX prodrug micelles possess remarkable biocompatibility, providing a strong foundation for subsequent experiments.

### In vitro release of the PSC/PTX micelles

To evaluate the in vitro release performance and redox sensitivity of drug-loaded micelles, the dynamic dialysis was employed. As shown in Fig. 4F, the particle size elevated from 102 to 110 nm at 24 h in the PBS solution (pH 7.4, 0 mM GSH). In contrast, the average size elevated from 102 to 269 nm (2-fold, *P* < 0.01) after 24 h in the pH 7.4 PBS solution (pH 7.4, 10 mM GSH). The above results demonstrated that 10 mM GSH can induce micellar drug release, confirming that PSC/PTX prodrug micelles enable precise drug release in response to the TME. The reduction-sensitive release of drug-loaded micelles was assessed in PBS (pH 7.4) supplemented at different GSH levels, simulating physiological and tumor-relevant redox conditions (Fig. 4G and H). When the concentration of GSH was elevated to 10 mM, the release rates of PTX and CUR from PSC/PTX prodrug micelles reached 91.3% and 90.7%, respectively, following a period of 48 h. This can be attributed to the high GSH concentrations facilitating the cleavage rate of disulfide bonds via thiol-disulfide exchange reactions, thereby accelerating the disintegration of micelles and enhancing drug release efficiency. In contrast, at low GSH concentrations (0 mM), the release rates were only 19.6% and 21.7% after 48 h. These results demonstrated that PSC/PTX prodrug micelles exhibit excellent reduction-sensitive characteristics. In the presence of high GSH concentrations, cleavage of disulfide bonds in PSC/PTX prodrug micelles leads to the separation of hydrophilic and hydrophobic groups, thereby facilitating the release of drugs encapsulated in the hydrophobic core.

### In vitro evaluation of antitumor efficacy

#### Drug resistance

The CCK-8 assay was applied to evaluate the drug resistance of SW480/PTX cells. The result indicated that the RI of SW480/PTX was determined as 10.43. Specifically, the IC_50_ value of SW480/PTX cells (43.52 ± 1.2 μg/ml) was 10.43-fold higher than that of SW480 cells (4.17 ± 0.3 μg/ml) (Fig. 4I and J). The SW480/PTX cells exhibited significant drug resistance, when RI > 5. Thus, SW480/PTX cells were confirmed to exhibit remarkable drug resistance.

#### Cytotoxicity assay

**Fig. 5.**
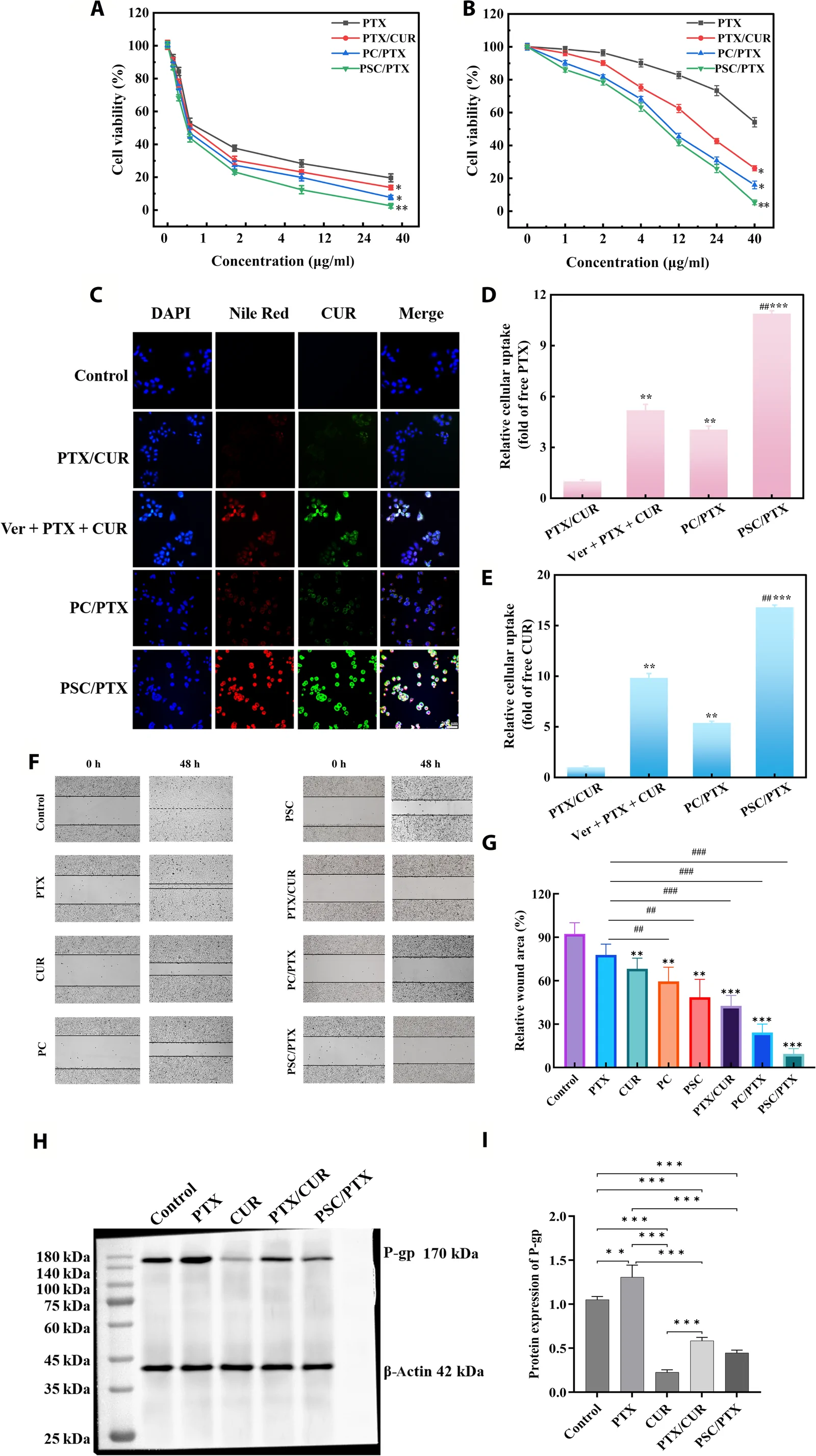
In vitro evaluation of the efficacy of anti-colon cancer agents. The cell survival rate of PTX, PTX/CUR, PC/PTX, and PSC/PTX in (A) SW480 and (B) SW480/PTX cells at different concentrations after incubation for 48 h at 37 °C (PTX concentration: 1 to 40 μg/ml) (*n* = 3, compared with PTX, *P < 0.05, **P < 0.01). (C) Cellular uptake of various drugs in SW480/PTX cells (scale bars: 100 μm) (cell nucleus: blue, CUR: green, PTX: red). Quantitative analysis of intracellular fluorescence intensity of (D) Nile Red (used as a PTX surrogate) and (E) CUR (*n* = 3, compared with PTX, **P < 0.01, ***P < 0.001; compared with verapamil treatment, ^##^*P* < 0.01; compared with CUR, **P < 0.01, ***P < 0.001; data were normalized to the corresponding free drug group, which was set as 1.0). (F) Representative images of SW480/PTX cell migration assays at 0 and 48 h after scratching, showing each treatment group (scale bar: 200 μm) and (G) the inhibitory effect of different treatment groups on wound healing rate of SW480/PTX cells (*n* = 3, compared with control, **P < 0.01, ***P < 0.001; compared with PTX, ^#^*P* < 0.05, ^##^*P* < 0.01, ^###^*P* < 0.001). Analysis of protein expression in SW480/PTX cells cultured under various drug treatment conditions was conducted using (H) Western blotting, (I) alongside the relative expression of the P-glycoprotein (*n* = 3, **P < 0.01, ***P < 0.001 compared to control group; ***P < 0.001 compared to PTX; ***P < 0.001 compared to CUR).

The in vitro cytotoxic potential of blank carriers (PC and PSC) and physical mixture (PTX/CUR) and drug-loaded micelles (PC/PTX and PSC/PTX) was assessed in NCM460, SW480, and SW480/PTX cells utilizing the CCK-8 assay (Fig. 4K to N). The blank carriers (PSC and PC) showed no obvious cytotoxicity against NCM460 cells (cell viability > 90%), confirming their favorable biocompatibility. Furthermore, the selectivity index (SI) of PSC/PTX, calculated as the ratio of the IC₅₀ value of NCM460 cells to that of SW480 cells (40.29/3.033), was 13.28, far exceeding the conventional safety threshold (SI > 3). This indicated that PSC/PTX exhibited potent tumor-selective cytotoxicity with low off-target toxicity toward normal colonic epithelial cells and afforded a broad therapeutic safety window. Following incubation with low concentrations of PC and PSC (1, 10, and 35 μM), the survival rates of SW480 and SW480/PTX cells showed no marked decrease, indicating that the blank carriers exerted minimal inhibitory effects on tumor cells at these concentrations. This result was attributed to the low CUR content in the blank carriers. Notably, the survival rates of SW480 and SW480/PTX cells exhibited a dose-dependent decline. Following the administration of elevated levels of PC and PSC (70 and 100 μM), cell survival rates decreased markedly. This reduction was attributed to the reduction-responsive properties of PSC, which enhanced CUR accumulation in tumor cells and potentiated inhibitory effects on both SW480 and SW480/PTX cells. Figure 5A and B illustrates the effects of PTX, PTX/CUR, PC/PTX, and PSC/PTX on cell survival. The survival rate of SW480/PTX cells decreased more slowly under PTX treatment compared to SW480 cells, indicating notable PTX resistance in SW480/PTX cells. Notably, the PSC/PTX group showed the strongest cytotoxicity among all tested groups. These results indicated that high concentrations of GSH within cells stimulate the cleavage of disulfide bonds in PSC/PTX prodrug micelles, leading to increased drug release and stronger toxic effects on SW480 and SW480/PTX cells.

#### Cellular uptake

As shown in Fig. 5C to E, PSC/PTX treatment significantly increased intracellular Nile Red (PTX surrogate) fluorescence by 10.89-fold compared to the free PTX group (*P* < 0.001), indicating markedly enhanced intracellular PTX accumulation. This effect was approximately 2-fold higher than that observed in the verapamil-treated group (5.19-fold), which served as a positive control for P-gp inhibition. This comparison confirms that PSC/PTX achieves functional inhibition of P-gp-mediated efflux that is at least as effective as the classical chemical inhibitor. Intracellular CUR fluorescence was enhanced by 16.81-fold relative to the free CUR group (*P* < 0.001), surpassing the effect of verapamil (9.83-fold). Collectively, these findings demonstrate that PSC/PTX effectively suppresses P-gp-mediated drug efflux, leading to markedly enhanced intracellular accumulation of both PTX and CUR.

#### Wound healing assay

The wound healing assay was employed to evaluate the inhibitory effect of PSC/PTX prodrug micelles on SW480/PTX cell migration (Fig. 5F and G). After 48 h of incubation, the control group exhibited nearly complete healing with 99% wound closure, while the PTX group showed 77.83% closure. This indicated that the PTX group exhibited the weakest inhibitory effect. Notably, the % wound closure of PSC/PTX group was 9.46% (*P* < 0.001). This could be attributed to the high concentration of GSH within tumor cells, which leads to the breaking of disulfide bonds, resulting in the release of the drug and the accumulation of CUR within the cells, thereby exhibiting a more pronounced inhibitory effect on tumor cell migration. Meanwhile, the synergistic effect of CUR and PTX not only enhanced the sensitivity of SW480/PTX cells to PTX but also improved the inhibitory effect on tumor cell migration. Therefore, PSC/PTX prodrug micelles possessed the strongest efficacy in inhibiting SW480/PTX cell migration.

#### Western blot

The P-gp expression levels in SW480/PTX cells were quantified using the Western blot assay. Figure 5H and Fig. S1 show an indicative set of blots, and Fig. 5I shows the corresponding quantitative plots. The PTX group demonstrated increased P-gp expression compared to the control. The overexpression of P-gp causes PTX to be expelled from tumor cells, resulting in a decrease in intracellular PTX concentration, thereby weakening the inhibitory effect of PTX on tumor cells and even triggering the tumor drug resistance. Conversely, the CUR group showed a notable 5.6-fold down-regulation of P-gp. CUR can down-regulate the activity of ornithine decarboxylase and inhibit polyamine synthesis, thereby reducing anaplerosis in the tricarboxylic acid (TCA) cycle and suppressing glutamine metabolism to decrease the levels of GSH and adenosine triphosphate (ATP) in SW480/PTX cells, ultimately reducing the drug efflux activity of P-gp [36]. The PSC/PTX group showed a 3.47-fold diminution in P-gp expression relative to the PTX group. These results suggested that the combination of CUR with PTX down-regulated P-gp expression and potentiated tumor cell sensitivity to PTX. Furthermore, GSH-mediated disruption of disulfide bonds in tumor cells facilitates drug release from micelles, thereby enhancing the inhibition of cell proliferation and improving antitumor efficacy.

#### Pharmacokinetics and biodistribution

The pharmacokinetic profiles of each drug were evaluated following intravenous administration as free monomers and PSC/PTX micelles, respectively. As shown in Tables S1 and S2, the PK study was initially conducted utilizing a noncompartmental model. Given the short half-life of PTX, which is 3.4 h, free drugs were swiftly eliminated from the bloodstream. In contrast, PSC/PTX micelles displayed an extended circulation half-life, with their *t*_1/2_ measured at 7.78 h. The PSC/PTX group displayed a considerably higher *C*_max_ than the free PTX group. Furthermore, the area under the plasma concentration–time curve from 0 to 48 h (AUC₀–₄₈) for each drug markedly improved when using PSC/PTX micelles in comparison to the respective monotherapy (AUC_0–48_, 16.73 versus 8.28 h·μg·ml^−1^ for PTX; 298 versus 117 h·μg·ml^−1^ for CUR). In conclusion, our study indicated that utilizing PSC/PTX micelles can enhance drug levels in the bloodstream, decrease drug clearance rates in vivo, prolong the duration drugs remain within the system, improve bioavailability, and elevate the effectiveness of cancer therapies.

**Fig. 6.**
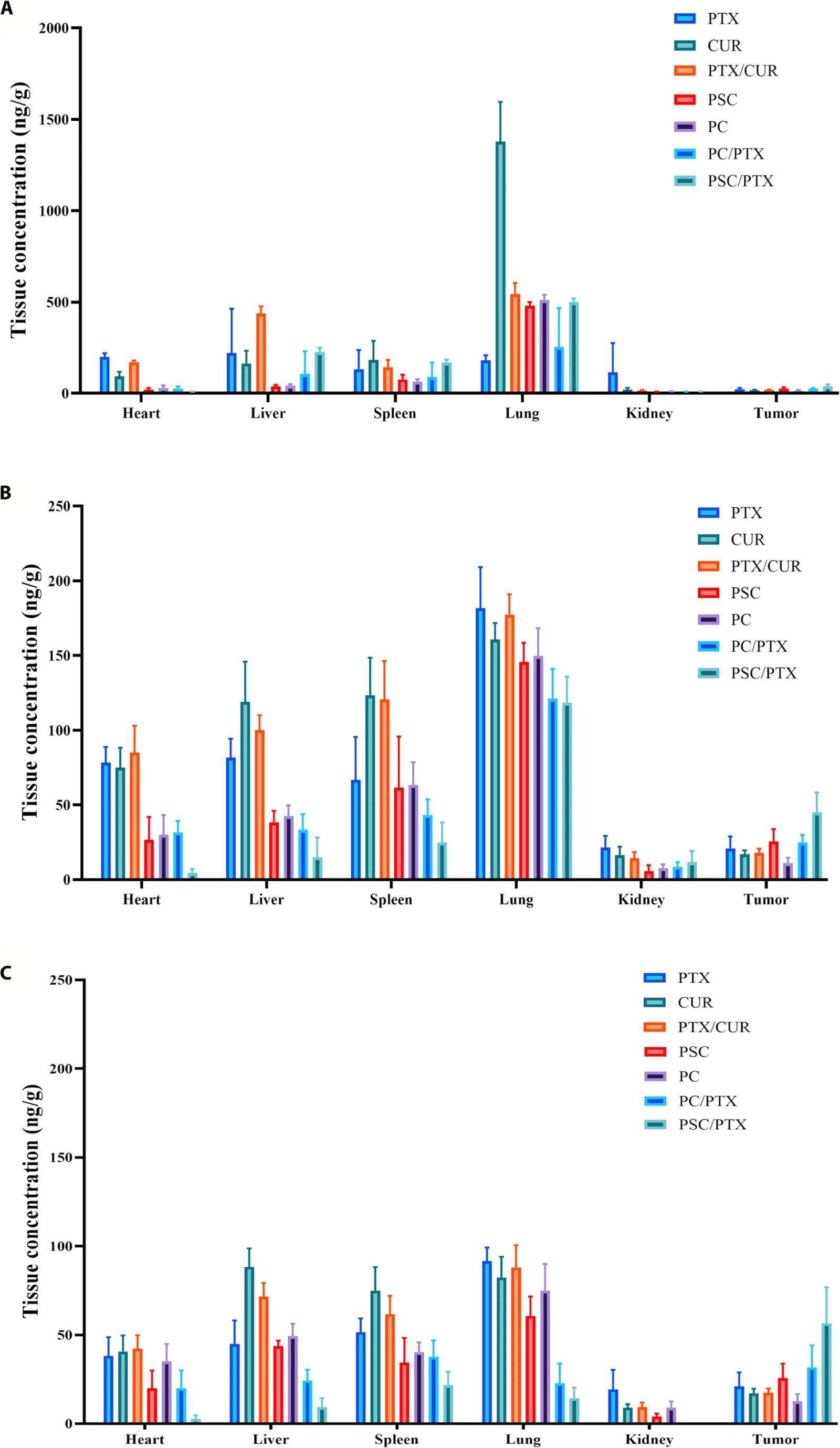
In vivo pharmacokinetic study. Biodistribution of each drug group in major organs and tumors at (A) 0.5 h, (B) 24 h, and (C) 48 h post-injection of each drug (means ± SD, *n* = 6).

As shown in Fig. 6A to C, following intravenous injection, PTX and CUR were rapidly distributed to other tissues. Notably, PTX and CUR exhibited higher accumulation in the liver, spleen, and lungs. Compared to the PTX or CUR monotherapy group, the drug-loaded micelles (PC, PSC, PC/PTX, and PSC/PTX) at 0.5 h post-administration were primarily distributed in the lungs and liver, with the CUR monotherapy group showing the highest lung accumulation. At 24 h post-administration, the levels of CUR and PTX in the liver and lungs decreased markedly (CUR: 25% decrease in lungs and 14% decrease in liver, PTX: 10% decrease in lungs and 9% in liver), and the PSC/PTX group exhibited the most pronounced reduction (liver: 15%, lungs: 23%). Furthermore, the accumulation level of PSC/PTX in tumor tissue was markedly elevated by 20% at 1 d post-administration relative to the PTX monotherapy group. At 48 h post-administration, the accumulation of PSC/PTX micelles in tumor tissue was further elevated by 10% (versus the 24-h time point of the same group), whereas their distribution in other normal organs (heart, liver, spleen, kidneys) was further reduced. This phenomenon can be linked to the distinct PEO–PPO–PEO block arrangement of P188. The outer PEO chains effectively avoid recognition and clearance by the mononuclear phagocyte system through steric hindrance effects. This property substantially prolongs the in vivo circulation time of encapsulated drugs, thereby enhancing their accumulation at tumor sites and reducing their distribution to other normal tissues.

**Fig. 7.**
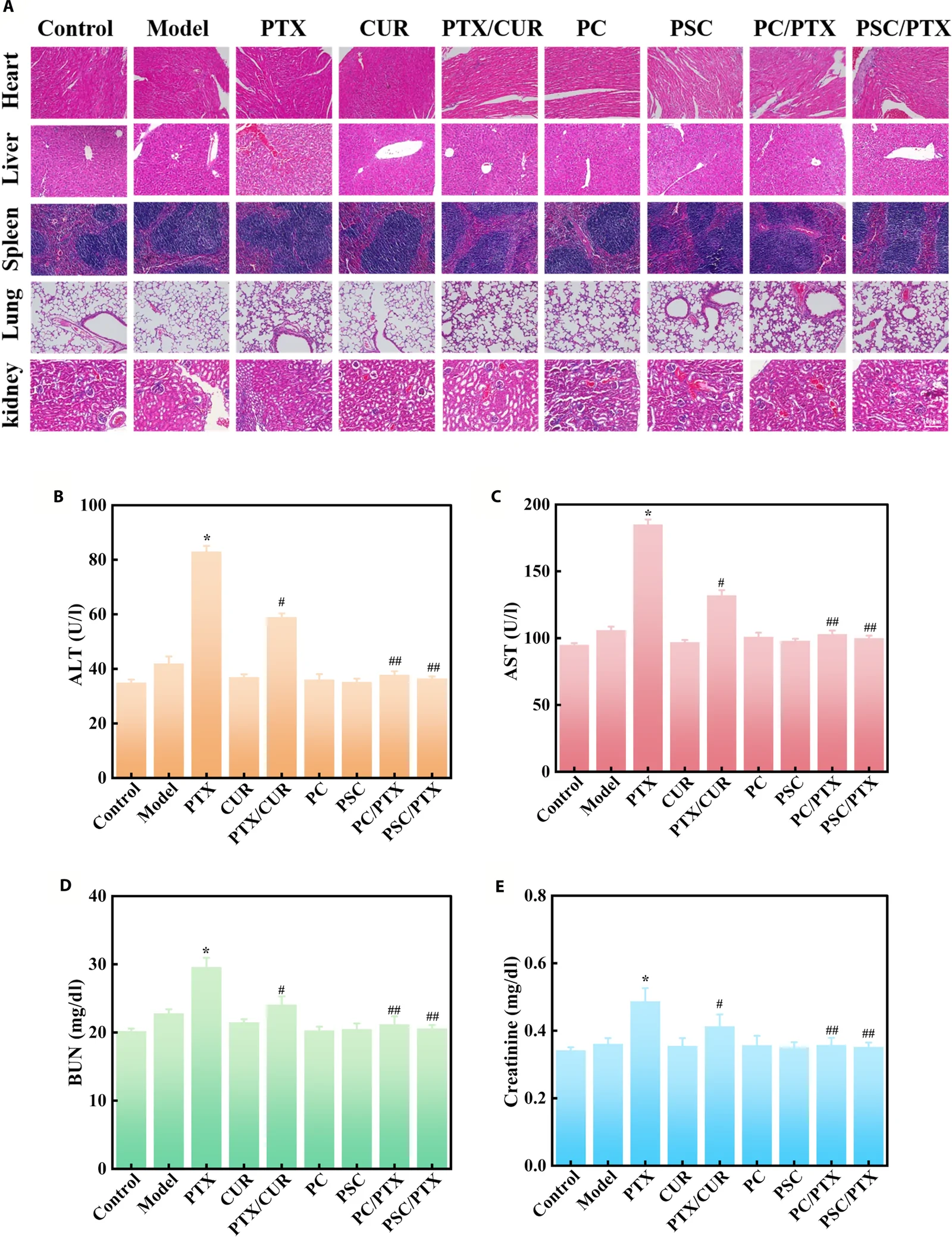
In vivo biocompatibility and safety evaluation of PSC/PTX. (A) Representative H&E-stained sections of major organs (heart, liver, spleen, lung, and kidney). Serum levels of (B) ALT, (C) AST, (D) BUN, and (E) Cr at the study endpoint. Data are presented as mean ± SD (*n* = 6, with comparison to control, *P < 0.05, **P < 0.01; compared with PTX, ^#^*P* < 0.05, ^##^*P* < 0.01).

As shown in Fig. 7A, H&E staining of kidney and liver sections from the free PTX group revealed marked pathological changes, including glomerular structural distortion, tubular disorganization with irregular lumens, epithelial degeneration, and interstitial hyperplasia with inflammatory infiltration in the kidneys, as well as hepatic disorganization, focal hemorrhage, sinusoidal congestion, and hepatocellular degeneration in the liver. In contrast, all other treatment groups exhibited essentially normal histomorphology without obvious pathological abnormalities, demonstrating favorable biosafety.

As shown in Fig. 7B to E, the free PTX group exhibited significantly elevated levels of serum AST, ALT, CREA, and BUN compared to the control group (*P* < 0.05), indicating that free PTX induced notable hepatotoxicity and nephrotoxicity. In contrast, treatment with PSC/PTX effectively reduced all 4 parameters to within normal ranges. Furthermore, the levels of these biomarkers in the control, model, PC/PTX, PC, and PSC groups all remained within the normal physiological ranges, with no statistically notable differences observed among these groups. Collectively, these results demonstrated that PSC/PTX effectively alleviated PTX-induced visceral toxicity. Moreover, intravenous administration of PSC/PTX formulations for 30 consecutive days did not elicit remarkable hepatic or renal toxicity, further verifying the favorable in vivo biocompatibility of the PSC/PTX.

### In vivo therapeutic efficacy

#### Animal model

**Fig. 8.**
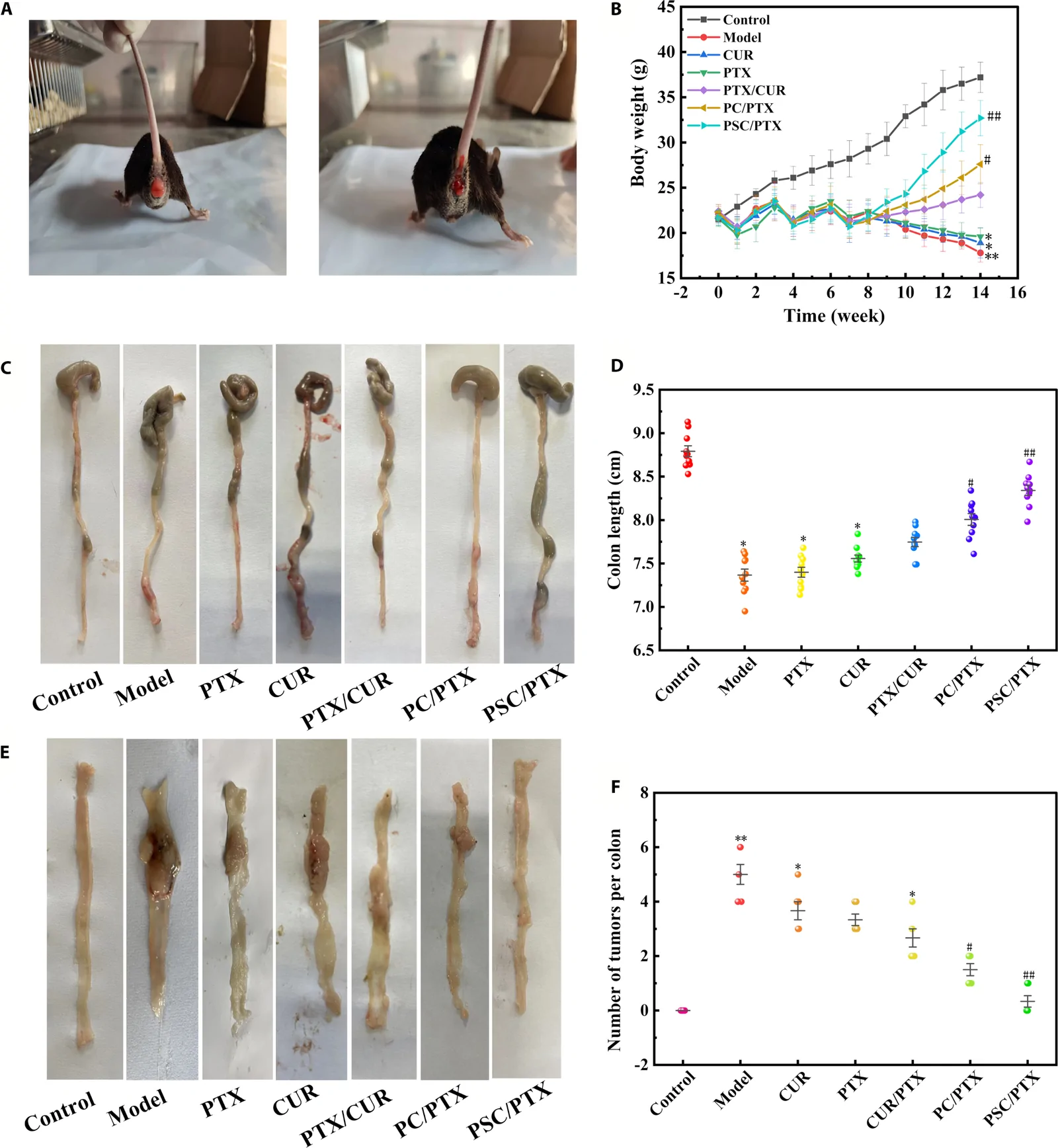
In vivo evaluation of the efficacy of anti-colon cancer. (A) Prolapse of the anus during the modeling of the model group. (B) Weight changes in AOM/DSS administered male C57BL/6 mice (*n* = 10, with comparison to control, *P < 0.05, **P < 0.01; compared with model, ^#^*P* < 0.05, ^##^*P* < 0.01). (C) Representative macroscopic morphology of colon tumors in each group and (D) comparison of colorectal length (*n* = 10; compared with control group, *P < 0.05; compared with model group, ^##^*P* < 0.01, ^#^*P* < 0.05). (E) Anatomy of the colorectum and (F) analysis of the number of tumors (*n* = 10; comparison with control group, *P < 0.05, **P < 0.01; compared with model group, ^#^*P* < 0.05, ^##^*P* < 0.01).

The results of mouse body weight, tumor volume, and tumor count recorded throughout the experiment were shown in Fig. 8A and B. The weight (control group) exhibited a progressively elevated trend throughout the experiment. After the administration of AOM injection, the remaining groups temporarily lost weight and then returned to normal except for the control group. However, after 3 cycles of 2% DSS administration, the model group displayed severe symptoms, including depression, notable weight reduction, hematochezia, anal prolapse, and an 87% decrease in weight compared to the control group (*P* < 0.01). Conversely, the administration groups (PTX, CUR, PTX/CUR, PC/PTX, PSC/PTX) showed varying levels of symptom alleviation. Importantly, the PSC/PTX group demonstrated the fastest recovery in weight, reaching 73% of the initial body weight by the endpoint (*P* < 0.01).

The severity of colorectal inflammation was evaluated using colon length and tumor count as primary endpoints (Fig. 8C to F). The colorectal length in the model group was markedly shortened. CUR monotherapy partially restored colorectal length (7.56 ± 0.13 cm versus model 7.37 ± 0.22 cm, *P* < 0.05). The above results demonstrated that PTX failed to exhibit anti-inflammatory activity against colorectal inflammation, whereas CUR demonstrated notable ameliorative effects. Relative to the model group, the colorectal length in the PTX/CUR group was 7.75 ± 0.17 cm, and that in the PC/PTX group was 8.01 ± 0.22 cm (*P* < 0.05) relative to the model group, suggesting that the combination of the 2 drugs markedly restored colon and rectal length. Particularly, the PSC/PTX group exhibited the most significant therapeutic effect (8.34 ± 0.19 cm, *P* < 0.01). Furthermore, tumors were clearly evident in the model group. PTX monotherapy significantly reduced the tumor count (3.33 ± 0.52, *P* < 0.05), indicating that PTX possessed potent antitumor activity. Similarly, the number of tumors decreased significantly in the PSC/PTX group (0.33 ± 0.52, *P* < 0.01). These findings demonstrated that PTX and CUR combination ameliorated colonic inflammation (restored colon length and attenuated weight loss) and potently suppressed tumorigenesis (minimal tumor count), achieving synergistic anti-inflammatory and antitumor effects.

#### H&E staining and pathology

**Fig. 9.**
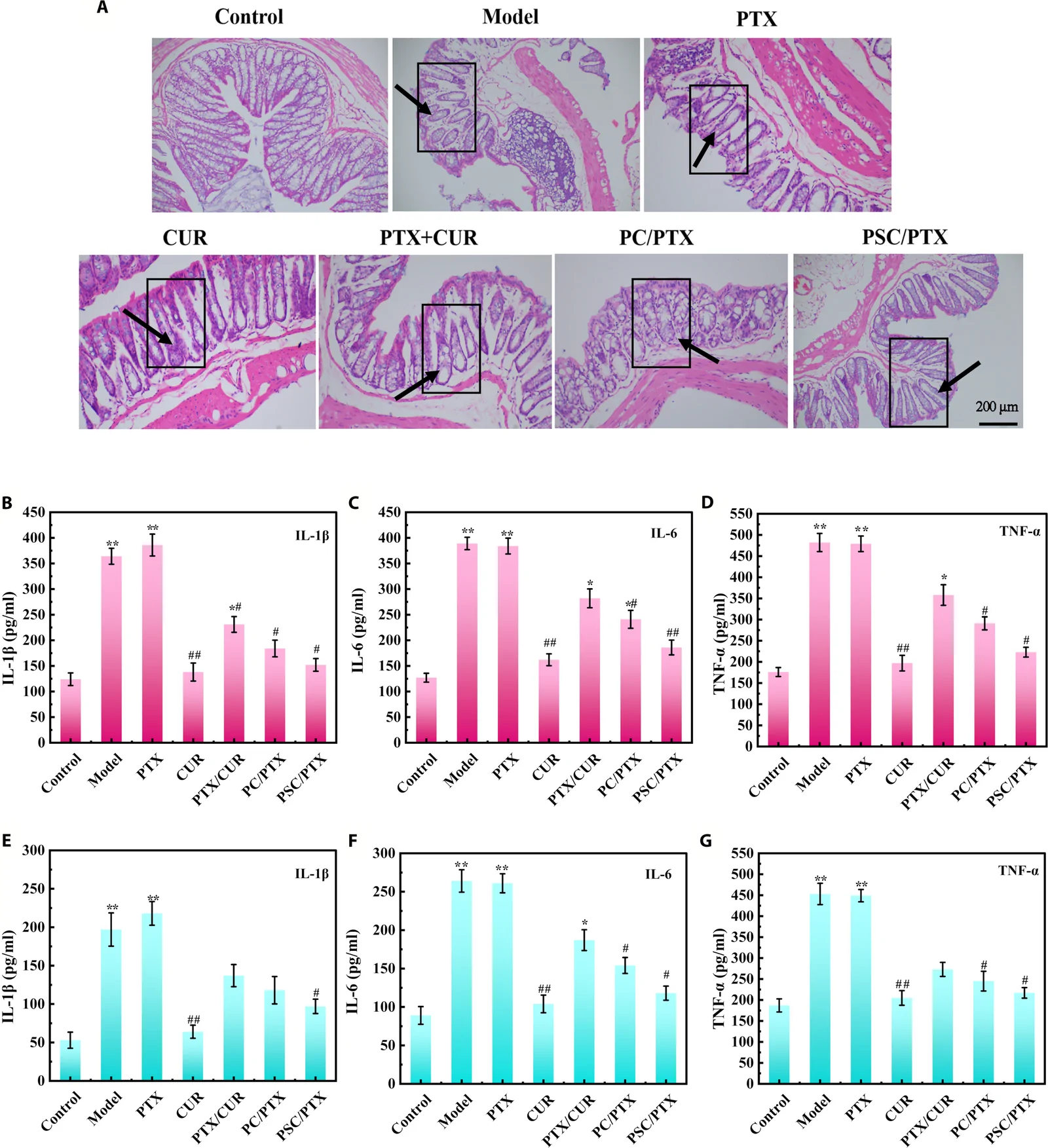
(A) Histological sections of small intestinal and rectal tissues stained with toluidine blue and eosin (scale bars: 200 μm). The expression levels of (B) IL-6, (C) IL-1β, and (D) TNF-α in serum (*n* = 5; compared with control group, *P < 0.05, **P < 0.01; compared with model group, ^#^*P* < 0.05, ^##^*P* < 0.01). The expression levels of (E) IL-6, (F) IL-1β, and (G) TNF-α in tissues (*n* = 5; compared with control group, *P < 0.05; compared with model group, ^#^*P* < 0.05, ^##^*P* < 0.01).

Histological analysis (Fig. 9A) revealed that the model group exhibited a disrupted epithelial mucus layer and an elevated nuclear-to-cytoplasmic ratio versus the control. While PTX and CUR monotherapy induced gradual repair, the PC/PTX and PSC/PTX combinations led to marked recovery of both the mucus layer and crypt structures. Additionally, the degree of inflammatory cell infiltration and gland disorganization was markedly reduced, with only a small number of goblet cells exhibiting slight alterations in nuclear division. Among all the groups, the PSC/PTX group showed the most pronounced effects, with tumor cells nearly absent, goblet cells largely restored, and the colorectal epithelial structure approaching normalcy. These results indicated that the PSC/PTX group more effectively alleviated damage inflicted by induced CRC on the mice colorectum, considerably enhanced the symptoms of CRC in mice, and achieved the synergistic therapeutic effects of anti-inflammatory and antitumor through intelligent combined drug delivery system.

#### ELISA assay

The expression levels of inflammatory factors (IL-6, IL-1β, and TNF-α) in serum and colorectal tissues in each group were determined by ELISA (Fig. 9B to G). The model group exhibited a notable up-regulation of TNF-α, IL-1β, and IL-6 in colorectal tissues and serum relative to the control group (*P* < 0.05). However, the PTX group exhibited an elevation in IL-1β levels in both colorectal tissue (218 ± 15.4 versus model 197 ± 21.6, *P* < 0.01) and serum (386 ± 21.4 versus model 364 ± 15.6, *P* < 0.01). No notable changes were detected in IL-6 and TNF-α. This could be ascribed to PTX, an established ligand for TLR4, which stimulates certain cells to produce IL-1β, subsequently initiating an inflammatory response [37]. The CUR group demonstrated potent anti-inflammatory properties relative to the model group, with significant decreases noted in the levels of IL-6, IL-1β, and TNF-α (all *P* < 0.01). Importantly, the most significant therapeutic effect was observed in the PSC/PTX group, which was associated with marked reductions in IL-6, IL-1β, and TNF-α levels in both serum and tissues compared to the model group (IL-6: *P* < 0.01; IL-1β and TNF-α: *P* < 0.05). These results suggested that CUR can reduce the elevated levels of IL-1β induced by PTX, indirectly inhibiting the inflammation triggered by PTX and mitigating its toxicity. The combination of PTX and CUR reduced the expression levels of inflammatory factors (TNF-α, IL-1β, and IL-6), leading to anti-inflammatory effects and ultimately inhibiting tumor growth. Therefore, the combination of the 2 drugs demonstrated a more potent effect against CRC.

### Intestinal microbiota

The gut microbiota is closely related to the occurrence and development of CRC. The occurrence of CRC is often accompanied by dysbiosis of the intestinal flora. This microbial community primarily consists of probiotic, neutral, and pathogenic bacteria. Probiotics (such as *Bacteroidetes, Eubacterium*, and *Bifidobacterium*) can inhibit the development of CRC. However, pathogenic bacteria (*Bacteroides fragilis*, *E. coli*, *Streptococcus*, *Enterococcus faecalis*, and *F. nucleatum*) can promote the development of CRC.

#### OTU

**Fig. 10.**
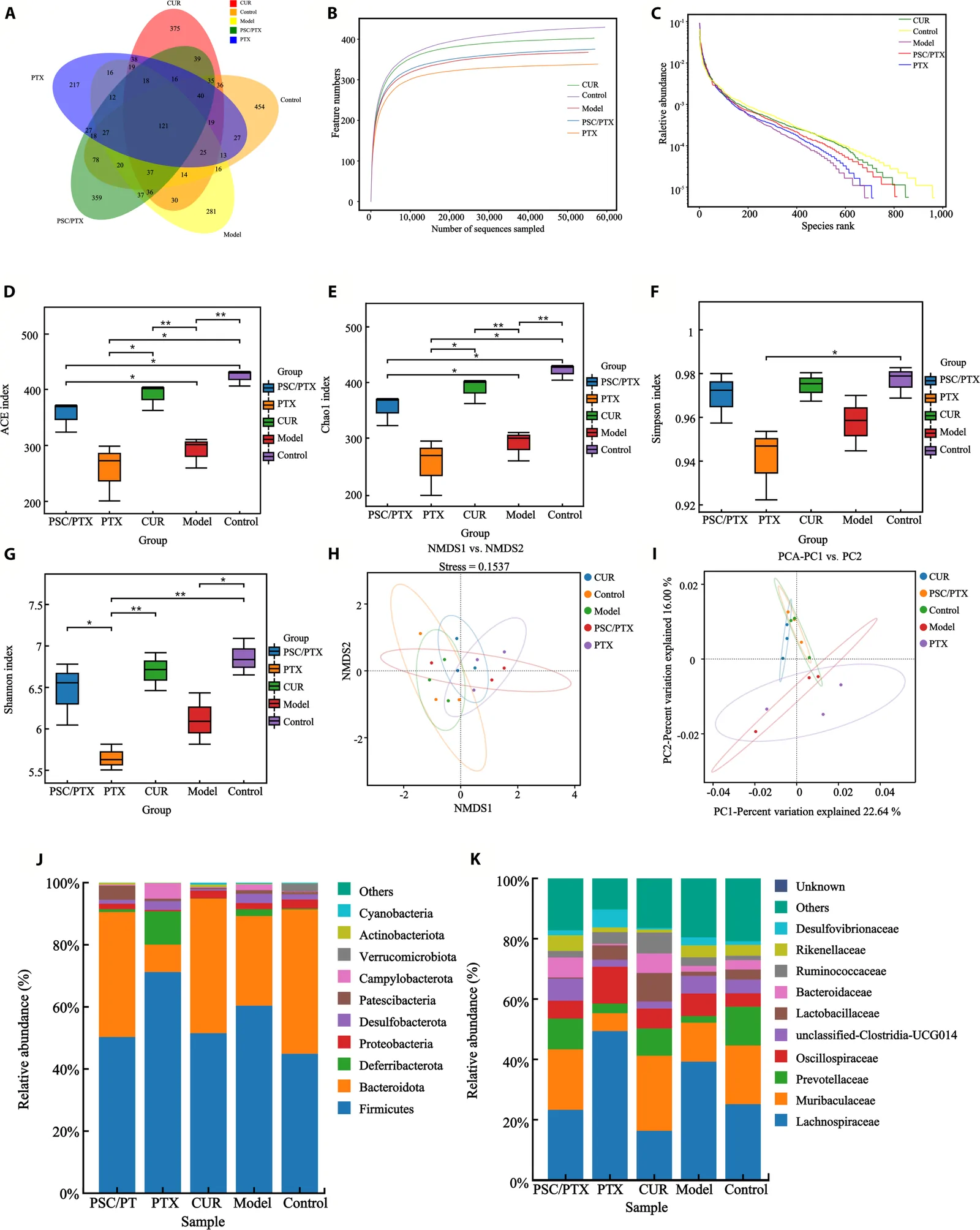
Study on the regulatory effect of PSC/PTX prodrug micelles on the intestinal microbiota. (A) OTU analysis. (B) Dilution curves. (C) Abundance distribution curves of gut microbiota. (D) Chao1 index, (E) ACE index, (F) Shannon index, and (G) Simpson index (*n* = 3; compared with control, *P < 0.05, **P < 0.01). (H) Nonmetric multidimensional scaling analysis and (I) principal components analysis (*n* = 3). Relative abundances of gut microbiota at (J) phylum and (K) family level (*n* = 3).

The comparative analysis of shared and unique operational taxonomic units (OTUs) among the various groups was shown by the Venn diagram (Fig. 10A). The model group demonstrated a significant reduction in OTU count in comparison to the control group (281 ± 0.54 versus 454 ± 0.17, *P* < 0.05), indicating that CRC diminished microbial diversity. The OTUs in the PTX group further decreased to 217, suggesting that PTX exacerbated microbiota dysbiosis. However, the PSC/PTX group restored OTUs to 359, approaching control levels. PTX disrupted gut homeostasis by suppressing beneficial probiotics (e.g., *Bifidobacterium*) and promoting pathogen bacteria (e.g., *E. coli*). In contrast, CUR ameliorated PTX-induced gut microbiota disruption, thereby indirectly enhancing the therapeutic efficacy of PTX.

#### Dilution and abundance distribution curves

Dilution and abundance distribution curves were utilized to evaluate the coverage of taxa at different sequencing depths to reflect species richness and diversity. As shown in Fig. 10B and C, PTX disrupted the composition of the intestinal microbiota. The abundance of intestinal microbiota elevated following the CUR treatment. Particularly, both the abundance and evenness of the gut microbiota markedly improved after the administration of PSC/PTX treatment, indicating that PSC/PTX prodrug micelles possessed the most potent therapeutic effect on gut microbiota. These results indicated that CUR alleviated the disruption of intestinal microbiota composition induced by PTX and mitigated toxic side effects of PTX, thereby exerting synergistic therapeutic effect in CRC treatment.

#### Alpha diversity and beta diversity

The intestinal microbiota of the 5 groups was characterized by analyzing alpha diversity, including richness [Chao1 and abundance-based coverage estimator (ACE) indices] and diversity/evenness (Shannon and Simpson indices) (Fig. 10D to G). The Chao1 index and ACE index in the model groups decreased notably (*P* < 0.01) compared to the control group, indicating that CRC disrupted gut microbiota homeostasis and decreased bacterial species abundance. Furthermore, the significant decrease in the Shannon index (*P* < 0.05) further indicated that CRC disrupted the compositional structure of gut microbiota and inhibited the development of microbial diversity. All 4 alpha diversity indices decreased after the administration of PTX, indicating that PTX could potentially perturb gut microbiota in CRC treatment. The PSC/PTX group showed a considerably increased level in the diversity and abundance of intestinal flora, indicating that the combination of CUR and PTX increased the diversity and abundance of intestinal flora, ameliorated the intestinal flora imbalance caused by colon cancer, and improved the comprehensive treatment effect against CRC.

To characterize the beta diversity of intestinal microbiota across these groups, nonmetric multidimensional scaling (NMDS) and principal components analysis (PCA) were employed (Fig. 10H and I). Based on NMDS analysis, the stress value was 0.1537. The analysis exhibited a high level of confidence, indicating effective separation of intestinal microbial community structure across groups. Meanwhile, PCA revealed that data points from the PTX group clustered closely with those of the model group, reflecting minimal compositional divergence. In contrast, the CUR and PSC/PTX groups clustered markedly closer to the control group. These exhibited that PTX was ineffective in ameliorating CRC-induced intestinal flora disruption. Conversely, the PSC/PTX prodrug micelle combination exhibited potent efficacy in restoring intestinal microbiota homeostasis, as evidenced by the near-complete recovery of gut microbiota composition to levels comparable with the control group.

#### Analysis of the structure of the intestinal flora

The regulatory effect of PSC/PTX prodrug micelles on gut microbiota was revealed by phylum level (Fig. 10J). The relative abundance of Firmicutes increased by 14% and 27% in the model and PTX groups, respectively, with corresponding 2-fold and 10-fold elevations in the Firmicutes/Bacteroidetes (F/B) ratio. Importantly, the PTX group exhibited a marked increase in *Desulfobacterota*, indicating that gut microbiota was disrupted by PTX and further promoted the occurrence of inflammation. In contrast, PSC/PTX prodrug micelles rebalanced the microbial structure by lowering *Firmicutes* and elevating *Bacteroidota* abundance, thereby normalizing the F/B ratio. Moreover, the PSC/PTX group showed a decrease in the level of *Desulfobacterota*, indicating that the combination of CUR and PTX improved the shortcomings of PTX on the destruction of intestinal flora and indirectly enhanced therapeutic efficacy.

The impact of prodrug micelles on gut microbiota was examined at the family level (Fig. 10K). Both the model and PTX groups exhibited a marked attenuation in the proportions of the *Muribaculaceae*, *Bacteroidaceae*, and *Prevotellaceae* families relative to the control group. This indicated that the disrupted abundance of intestinal beneficial commensal bacteria led to intestinal microbiota dysbiosis, which in turn indirectly facilitated CRC progression. In the state of intestinal dysbiosis, conditional pathogenic bacteria (such as *F. nucleatum*) trigger the release of inflammatory factor (such as IL-6, TNF-α, and IL-1β), thereby promoting the development of CRC [38]. Notably, the proportion of probiotics (*Muribaculaceae*, *Bacteroidaceae*, and *Prevotellaceae*) markedly increased after treatment with PSC/PTX. These results demonstrated that the combination of CUR and PTX mitigated the destruction of probiotics (*Muribaculaceae*, *Bacteroidaceae*, and *Prevotellaceae*) by PTX, regulated the gut microbiota, and reduced the release of inflammatory factors, thereby indirectly enhancing its efficacy in CRC treatment.

### Immunohistoanalysis

As shown in Fig. S2A, a markedly reduced CD8^+^ T cell count was detected in the model group, while the CD8^+^ T cell counts increased to varying degrees in all other groups. Notably, the PSC/PTX group exhibited a markedly elevated level in CD8^+^ T cell density. This could be attributed to PTX-induced immunogenic cell death (ICD), which triggered tumor cells to release antigens and damage-associated molecular patterns (DAMPs), thereby activating dendritic cells (DCs). These activated DCs then present antigens and deliver costimulatory signals, driving the proliferation and differentiation of CD8^+^ T cells into effector T cells, and ultimately leading to a substantial elevation in CD8^+^ T cell density [39]. CUR effectively reversed immunosuppression of CD8^+^ T cells by antagonizing the NF-κB/signal transducer and activator of transcription (STAT3) signaling pathway and down-regulating proinflammatory factor expression while simultaneously attenuating myeloid-derived suppressor cell function, thereby promoting CD8^+^ T cell proliferation and activation [40].

Tregs, characterized by their crucial transcription factor Foxp3^+^, facilitate tumor immune evasion by inhibiting effector T cell activity. The model group showed an increased percentage of Foxp3^+^ Tregs, indicating the existence of an immunosuppressive environment within the TME (Fig. S2B). PTX enhances the activity of antigen-presenting cells (APCs) and promotes the activation and proliferation of effector T cells [41]. CUR down-regulates the expression of Foxp3^+^ Tregs, disrupts its intracellular stability, and inhibits Treg clonal proliferation. When combined, PTX and CUR exert a potent synergistic therapeutic effect through cooperative modulation of the tumor immune microenvironment. PTX effectively activates the DC–CD8^+^ T cell immune axis, while CUR alleviates immunosuppression by inhibiting Treg differentiation and function, resulting in complementary immunomodulatory effects. Consequently, the PSC/PTX group exhibited the lowest proportion of Tregs, indicating that PSC/PTX possesses the strongest inhibitory effect.

The model group displayed a reduced density of CD86^+^ cells, as illustrated in Fig. S2C, with a prevalence of M2-type (CD206^+^) cells, indicating an immunosuppressive microenvironment. The CD86^+^ macrophage density in the monotherapy group exhibited a slight elevation but with a relatively scattered spatial distribution, suggesting the limited efficacy and insufficient targeting ability of monotherapy. The PSC/PTX group exhibited a higher density of CD86^+^ cells with a more concentrated distribution, which may be related to GSH-responsive drug release. Concurrently, PTX activates the TLR4 receptor on macrophage surfaces, thereby initiating the NF-κB pathway. This promotes nuclear shuttling of the transcription factor, which activates the M1-type gene transcription program [42]. Consequently, CD86^+^ expression is up-regulated, and characteristic proinflammatory factors including IL-12 and TNF-α are secreted, ultimately driving the transition to the M1 phenotype. CUR alleviates the suppression of M1 polarization in the TME by inhibiting STAT3-mediated immunosuppressive signaling pathways. The combination of the 2 drugs synergistically promotes macrophage polarization toward the M1 phenotype [43]. Furthermore, CUR exerted anti-inflammatory effects and modulated the gut microbiota, thereby improving the tumor immune microenvironment. Together, these effects form a multifaceted, multidimensional therapeutic system encompassing anti-inflammatory–antitumor–gut microbiota–regulation–immune modulation. This multi-target synergistic mechanism not only markedly enhances the antitumor immune response but also provides a new strategic direction for combination therapy in CRC.

An immunosuppressive, tumor-promoting microenvironment was evidenced in the model group by immunofluorescence analysis (Fig. S2D), which showed a higher density of CD68^+^ macrophages with a predominantly M2 (CD206) phenotype. Notably, a markedly increased M1/M2 ratio was observed in the PSC/PTX group (CD86^+^↑, CD206^+^↓), suggesting that the combination of the 2 drugs produced a stronger synergistic effect. Specifically, PTX facilitates a shift toward the M1 phenotype by activating the NF-κB/STAT1 signaling pathway to up-regulate the M1 marker CD86^+^ while concurrently down-regulating the M2 marker CD206^+^ by inhibiting key signaling pathways driving M2 polarization [e.g., phosphatidylinositol 3-kinase (PI3K)/Akt/STAT3] [44]. The CUR facilitated the transformation of macrophages into the M1 phenotype by blocking the STAT3/NF-κB pathway and decreasing the production of proinflammatory cytokines, including IL-6 and IL-1β [45]. This bidirectional regulation markedly increases the proportion of CD86^+^ cells while decreasing CD206^+^ cells, ultimately achieving a substantial shift in M1/M2 phenotype balance toward the antitumor M1 phenotype. Furthermore, this polarization-modulating effect is substantially superior to that of either monotherapy group, laying a solid basis for potentiating antitumor immune responses mediated by M1-type antitumor immunity.

## Conclusion

In this study, we constructed an intelligent, reduction-responsive prodrug micellar nanosystem (PSC/PTX). By integrating prodrug conjugation and physical encapsulation into a novel hybrid strategy, this system enables precise co-delivery and TME-responsive release of CUR and PTX, thereby ensuring synergistic action at the target site. In vitro studies demonstrated that the PSC/PTX prodrug micelles achieve TME-responsive drug release via disulfide bond cleavage. Simultaneously, they down-regulated P-gp expression to reverse drug efflux and inhibited the secretion of inflammatory factors, thereby simultaneously addressing multidrug resistance and the pro-tumorigenic inflammatory milieu. This dual mechanism effectively enhances the intracellular accumulation of both CUR and PTX, addressing key limitations of PTX monotherapy. Critically, this nanosystem fundamentally altered the in vivo fate of the encapsulated drugs. Pharmacokinetically, the PSC/PTX nanosystem notably prolonged systemic circulation and enhanced the bioavailability of both drugs. This optimization led to superior tumor-targeting, with sustained drug accumulation within tumor tissues and reduced distribution in nontarget tissues. In the inflammation-driven CRC model, PSC/PTX exhibited remarkable antitumor efficacy, directly reducing tumor growth and restoring colonic architecture. Concurrently, it mediated therapeutic remodeling of the gut–immune axis, restoring microbiota homeostasis, quenching inflammation, and reprogramming the immunosuppressive TME.

Collectively, this work has established a novel combinatorial strategy that concurrently targets the drug delivery–microbiota–immunity axis and overcomes resistance. By disrupting key nodes in the vicious cycle of inflammation-driven carcinogenesis, it presents a potent and innovative nanotherapeutic paradigm for refractory CRC. Future studies will focus on evaluating long-term biosafety, validating efficacy in patient-derived organoids, and exploring synergistic potential with immune checkpoint inhibitors to further augment antitumor immunity, thereby connecting the divide between preclinical assessments and clinical applications.

### Discussion

Although we have demonstrated in vitro that PSC/PTX reduces PTX efflux, and the AOM/DSS model reliably recapitulates inflammation-driven colorectal carcinogenesis, this model does not inherently possess PTX resistance and therefore cannot fully mimic the resistant microenvironment. This discrepancy constitutes a clear limitation of our study. Future work will need to establish a PTX-resistant CRC xenograft or genetically engineered mouse model to better validate the therapeutic efficacy of PSC/PTX in overcoming drug resistance in vivo. Despite these limitations, our findings still provide a promising new direction for the development of nanoparticle drug delivery systems to reverse PTX resistance in CRC, and lay a preliminary experimental foundation for subsequent translational research.

## Data Availability

The data that support the findings of this study are available from the corresponding author upon reasonable request.
