# Supplementary material for: A Glutathione-Responsive Prodrug Nanosystem for Colorectal Cancer: Synergistic Co-Delivery of Paclitaxel and Curcumin to Combat Multidrug Resistance and Reinvent the Immunosuppressive Microenvironment
Source: Biomater Res. 2026 Sep 17;30:0399. doi: 10.34133/bmr.0399 (PMC13583130; doi:10.34133/bmr.0399)
Supplement: Supplementary file 1 [file bmr.0399.f1.docx]

**Figure S1.** Uncropped full-length western blot immunoblotting images.

**Table S1**

**Pharmacokinetic parameters for PTX were assessed through the administration of PTX and PSC/PTX micelles (mean ± SD, n = 6, *^*^p* < 0.01)**

| Parameters |  | PTX group |  | PSC/PTX group |
| --- | --- | --- | --- | --- |
| AUC_0–48_ (h^.^μg mL^−1^) |  | 8.28 ± 2.32 |  | 16.73 ± 3.79^*^ |
| MRT (h) |  | 1.37 ± 0.48 |  | 7.82 ± 1.15* |
| C_max_ (μg^.^mL^−1^) |  | 6.24 ± 0.32 |  | 7.58 ± 0.59 |
| t_1/2_ (h) |  | 3.4 ± 1.15 |  | 7.79 ± 2.47^*^ |
| CL (L h^− 1^ kg^−1^) |  | 0.91 ± 0.035 |  | 0.79 ± 0.01 |
| Vd (L^.^kg^− 1^) |  | 2.5 ± 1.03 |  | 4.5 ± 2.12 |

**(vs PTX, ^*^*p* < 0.01)**

**Table S2**

**Pharmacokinetic parameters of CUR were assessed through the administration of CUR and PSC/ PTX micelles (mean ± SD, n = 6, ^#^*p* < 0.01).**

| Parameters |  | CUR group |  | PSC/PTX group |
| --- | --- | --- | --- | --- |
| AUC_0–48_ (h^.^μg mL^−1^) |  | 117 ± 18.67 |  | 298 ± 13.56**^#^** |
| MRT (h) |  | 8.34 ± 0.25 |  | 18.14 ± 0.65 |
| C_max_ (μg^.^mL^−1^) |  | 5.1 ± 1.27 |  | 13 ± 3.27 |
| t_1/2_ (h) |  | 0.56 ± 0.17 |  | 5.89 ± 0.21 |
| CL (L h^− 1^ kg^−1^) |  | 0.085 ± 0.42 |  | 0.033 ± 0.27 |
| Vd (L^.^kg^− 1^) |  | 0.8 ± 0.12 |  | 6.1 ± 1.25 |

**(vs CUR, ^#^*p* < 0.01)**

**Figure S2.** Immunohistochemical analysis. (A) Immunofluorescence staining for CD8⁺ T cells (green) and DAPI (blue) in CRC tissues. Scale bar: 100 μm. (B) Immunofluorescence staining for FoxP3⁺ Tregs (red) and DAPI (blue). Scale bar: 100 μm. (C) Co-localization analysis via immunofluorescence staining for CD68⁺ (red), CD86⁺ (M1, green), and DAPI (blue). Scale bar: 100 μm. (D) Immunofluorescence staining for CD68⁺ (total macrophages, red), CD206⁺ (M2, green), and DAPI (blue) to assess M1/M2 polarization. Scale bar: 100 μm.
